# Identification of the *Ilex macrocarpa* anthracnose pathogen and the antifungal potential of the cell-free supernatant of *Bacillus velezensis* against *Colletotrichum fioriniae*

**DOI:** 10.3389/fmicb.2024.1419436

**Published:** 2024-06-20

**Authors:** Chun Fu, Shushan Wan, Peng Yang, Xizhu Zhao, Yueyao Yan, Shijiao Jiang, Habib Ali

**Affiliations:** ^1^Key Laboratory of Sichuan Province for Bamboo Pests Control and Resource Development, Leshan Normal University, Leshan, China; ^2^Key Laboratory of Southwest China Wildlife Resources Conservation, College of Life Science, China West Normal University, Nanchong, China; ^3^Department of Agricultural Engineering, Khwaja Fareed University of Engineering and Information Technology, Rahim Yar Khan, Pakistan

**Keywords:** anthracnose, *Colletotrichum fioriniae*, *Bacillus velezensis*, biocontrol, *Ilex macrocarpa*

## Abstract

**Introduction:**

Anthracnose is a significant fungal disease that affects tree growth and development, with Colletotrichum spp. exhibiting host non-specificity and targeting various organs, making disease control challenging.

**Methods:**

This study aimed to identify the pathogenic species causing anthracnose in *Ilex macrocarpa* in Nanchong, Sichuan Province, and screen effective fungicides, particularly biological ones. The pathogen was identified as *Colletotrichum fioriniae* through morphological observation, pathogenicity assays, and molecular biological methods. Three biological and five chemical fungicides were evaluated for their effects on the mycelial growth and spore germination rate of the pathogen.

**Results:**

The results indicated that prochloraz was the most effective chemical fungicide, while the cell-free supernatant (CFS) of *Bacillus velezensis* had the most significant inhibitory effect among the biological fungicides. Transcriptome analysis revealed that the CFS of *B. velezensis* significantly reduced the expression of genes associated with ribosomes, genetic information processing, membrane lipid metabolism, and sphingolipid biosynthesis in *C. fioriniae*. Additionally, the glutathione pathway’s expression of various genes, including key genes such as GST, GFA, Grx, TRR, and POD, was induced. Furthermore, the expression of 17 MFS transporters and 9 ABC transporters was increased. Autophagy-related ATGs were also affected by the *B. velezensis* CFS.

**Discussion:**

These findings suggest that the *B. velezensis* CFS may inhibit *C. fioriniae* through interference with ribosomes, genetic information processing, cell membrane metabolism, and energy metabolism. These results provide potential target genes for the *B. velezensis* CFS and insights into the antifungal mechanism by which *B. velezensis* inhibits *C. fioriniae*.

## Introduction

The genus Ilex belongs to the monogeneric family Aquifoleaceae and consists of more than 500 accepted species distributed in regions with mesic growing conditions worldwide, with the highest biodiversity observed in South America and Asia (Geukens et al., 2023). In China, members of this genus grow naturally in various forest ecosystems, with most populations exhibiting wide morphological variation and strong adaptability. These species have been extensively used for breeding and introduced multiple times to other parts of the world. *Ilex macrocarpa* Oliv. is a deciduous tree belonging to the genus Ilex and is found primarily in southern China. *I. macrocarpa*, a forest tree species with high application value, is utilized for its ornamental properties and medicinal value, as well as for construction or furniture production (Wang et al., 2023). Colletotrichum spp. are important pathogenic fungi that compose a large family with a wide variety of species worldwide, and 16 species complexes have been reported globally. The pathogenicity characteristics and virulence of these fungi vary among hosts, so accurate identification of these species is crucial in the control process (Bordoh et al., 2020). Moreover, *Colletotrichum* spp. have a broad range of hosts, including cash crops, fruit trees, vegetables, and other plants. In addition, *Colletotrichum* spp. can infect flowers, fruit, stems, leaves, and other plant organs (Hu S. et al., 2022; Salotti et al., 2022). Therefore, the nonspecific host and diverse types of infected organs of *Colletotrichum* spp. increase the difficulty of disease control, resulting in substantial agricultural and horticultural losses (Veloso et al., 2021). Although studies have focused on anthracnose in agriculture and horticulture, and the application of chemical pesticides has been an effective way to control this disease, studies accurately identifying the pathogen that causes anthracnose in forest trees and performing targeted control are limited (Gao et al., 2020; Ntui et al., 2021). However, due to the special requirements of forest ecosystems for environmental safety, chemical pesticides must be used cautiously, and more sustainable and safer alternative methods are urgently needed.

The bacterial genus *Bacillus* has been studied for its high capacity to produce a vast array of secondary metabolites with antagonistic activities and plays a significant role in biocontrol against many phytopathogens, including some species of *Aspergillus*, *Penicillium* and *Ralstonia*, as well as species such as *Botrytis cinereal* (Ahmed et al., 2022; Song et al., 2022; Dobrzyński et al., 2023). Previous studies reported that some antagonistic compounds produced by *Bacillus* spp. in liquid culture, including iturins, a bacteriocin, a peptide lipid surfactant, and antimicrobial peptides, are the main factors that inhibit fungal growth (Khan et al., 2021). In addition, the cell-free supernatant (CFS) of *B. velezensis* exhibited a potent antifungal effect by inhibiting spore germination, germ tube elongation and hyphal growth of *B. cinerea* and *Penicillium olsonii in vitro* and could damage the mycelial cells by promoting excessive accumulation of ROS to realize its biological control function (Zhao et al., 2022; Zhang et al., 2023). However, as *Bacillus* spp. are potential biocontrol bacteria, the antagonistic mechanism of *Bacillus* spp. against pathogens needs further investigation.

To identify the causative pathogen of *I. macrocarpa* anthracnose in Nanchong, China, in this study, the pathogen was isolated from diseased plants. The pathogenicity of the isolates was determined through Koch’s postulates, and the pathogen species was identified by combining morphological observations and molecular biological analyses. To identify effective control agents, the present study included an investigation of the inhibitory effects of common fungicides and selected promising biocontrol bacteria. The mechanism underlying the inhibition of *Colletotrichum fioriniae* by the CFS of *Bacillus velezensis* was also examined by transcriptome analysis. The aim of this study was to provide an important theoretical basis for the effective prevention and control of holly anthracnose disease in Nanchong, China.

## Materials and methods

### Isolation and characterization of the pathogen

Samples of *I. macrocarpa* leaves with typical anthracnose symptoms were collected from an artificial forest in Nanchong, Sichuan, China (EN), in April 2021 and 2022. The samples were cut into approximately 5 × 5 mm pieces at the juncture of diseased and healthy areas, and the surfaces were disinfected with 75% ethanol solution for 30 s and then with 3% sodium hypochlorite for 2 min. This was followed by rinsing with sterile distilled water 3 times and drying with sterilized filter paper. and the samples were then placed onto potato dextrose agar (PDA; 20% diced potato, 2% glucose, and 1.8% agar) medium for cultivation in the dark at 25°C. Single colonies were isolated using the procedures developed by Chomnunti et al. (2014). Colonies were grouped according to morphology, and all isolates were observed microscopically to select representative isolates.

### Pathogenicity tests

Subsequent experiments were carried out on detached leaves to confirm the pathogenicity of the representative isolates. The surfaces of healthy leaves were wiped with 75% ethanol and rinsed repeatedly in sterile water. After drying on sterile filter paper, the leaves were wounded by pricking with a sterile needle. Plugs (5 mm) were removed from the edges of the isolates, and the side with mycelia was attached to the wound, while the control had PDA plugs of the same size attached. The inoculated leaves were covered with plastic film, observed once a day, placed in sterile Petri dishes containing moistened filter paper and incubated at 28 ± 2°C for 7 days. The isolate that exhibited similar typical anthracnose symptoms after inoculation was selected, and the strain was reisolated from inoculated leaves according to the procedures described in Section 2.1, thus conforming to Koch’s postulates and confirming the pathogenicity of the strain.

### Morphological identification and phylogenetic analyses

Plugs of the isolates were inoculated into the center of the PDA plates and incubated for 7 days at 28°C under a 12/12 h light/dark cycle, after which the colony color, texture, and hyphal morphology were observed and recorded. The conidia and appressoria were observed, described, and measured using a light microscope (DM500, Leica, Wetzlar, Germany) (*n* = 30).

To conduct phylogenetic analyses, genomic DNA was extracted from 0.5 g of fresh hyphae using a DNA extraction kit (TaKaRa Bioengineering Co., Ltd., Dalian, China) and stored at −20°C. The internal transcribed spacer (*ITS*), histone 3 (*HIS3*), chitin synthase (*CHS-1*), actin (*ACT*), β-tubulin (*TUB2*) and glyceraldehyde-3-phosphate dehydrogenase (*GAPDH*) regions were amplified as described in Weir et al. (2012). The primers used, along with their respective sequences, are presented in Supplementary Table S1. PCR product sequencing was completed by Quintara Company (Wuhan, China).

The sequences were aligned using BLAST in the NCBI database. The homology was also analyzed via BLAST, and the resulting sequences were deposited in GenBank. All sequences used for the phylogenetic analyses in this study are listed in Supplementary Table S2. The six sequences were individually aligned to the locus-based reference sequences using MEGA7.0. Phylosuite V1.2.2 was used to concatenate the six aligned sequences, and then Partition Finder 2 was used to select the best fit models for the multilocus phylogenetic analyses. MrBayes in Phylosuite was used for Bayesian inference (2 parallel runs, 2,000,000 generations), and the top 25% of the sampled data was discarded. IQtree V 1.6.8 was used to establish and adjust the phylogenetic tree according to the maximum likelihood (ML) method. *C. orchidophilum* (CBS 632.80) was used as an outgroup. ML analysis under the edge-linked partition model for 1,000 standard bootstraps.

### Fungicide exposure treatment

The chemical fungicides used in this study were 50% carbendazim wettable powder (Wuhan Woxuan Technology Co., Ltd., China), the analytical standard prochloraz (Merck KGaA, Darmstadt, Germany), 95% paclobutrazol wettable powder (Shanghai Macklin Inc., China), the analytical standard thiophanate-methyl (Shanghai Yuanye Biology Co., Ltd., China), and the analytical standard imazalil (Shanghai Yuanye Biology Co., Ltd., China). Five different chemical reagents were dissolved in dimethyl sulfoxide (DMSO) to prepare a high-concentration mother liquor, which was then filtered through a Millex-HV filter (0.22 mm, 13 mm diameter; Millipore, Billerica, MA, United States) to obtain the sterile mother liquor. The mother liquor was diluted to 0 mg/L, 0.05 mg/L, 0.1 mg/L, 0.5 mg/L, and 1 mg/L in PDA for exposure treatment.

The biological fungicides used in this study were *Bacillus subtilis* ATCC6633 and *B. velezensis* SB023. One milliliter of cell suspension was added to 100 mL of nutrient broth (NB; 20 g of glucose, 5 g of yeast extract, 10 g of peptone, and 5 g of NaCl, pH 6.5–6.7), incubated for 48 h at 28°C with shaking at 200 rpm, and then adjusted to approximately 1 × 10^8^ CFU/mL, as measured with a hemocytometer. The culture was centrifuged at 4,000 × *g* for 15 min and then filtered through a Millex-HV filter to obtain the sterile CFS. The CFS was diluted to 0, 3, 5, 8, and 10% (v/v) in PDA for exposure treatment.

A mycelial plug of the pathogen was collected by a sterilized punch with a diameter of 0.5 cm and placed in the center of each reagent-amended medium. All medium containing plates were incubated at 28°C in the dark. The cross method was applied to measure the diameter of the colony, and the growth inhibition rate was calculated as the follows: rate of growth inhibition (%) = (colony diameter of control - colony diameter of treatment/- colony diameter of control) × 100%.

### Transcriptome analysis

Total RNA was extracted using a fungal RNA kit (Omega Bio-Tek Inc., Norcross, GA, United States). cDNA libraries with an average insert size of 380 bp were prepared using an Illumina mRNA-seq sample preparation kit and sequenced and analyzed (by Wuhan SeqHealth Tech Co., Ltd., Wuhan, China) in accordance with the Illumina standard protocol. The RNA-sequencing (RNA-seq) reads were aligned to the WHG5 genome sequence assembled in previous research.

All read alignment positions of each paired-end read were mapped against the genome sequence and exon splicing junctions using HISAT2 software with the BWT algorithm. Read counts per gene were determined from the primary read alignments using HTSeq, and the read count files with FPKM > 1 were analyzed with the “DESeq2” R package to identify differentially expressed genes (DEGs). An absolute log2-fold change > 1 and statistical significance (*p* value < 0.05) were used as the criteria to identify the DEGs. GO terms with a false discovery rate (*p* value after correction) ≤ 0.05 were defined as significantly enriched according to the hypergeometric test. The selected DEGs were categorized according to the protein functions predicted by the Kyoto Encyclopedia of Genes and Genomes (KEGG) database, after which the number of genes in each pathway was calculated. The KEGG pathways with significant enrichment of DEGs compared with the genomic background were defined using the hypergeometric test under the same conditions.

### Quantitative real-time PCR analysis

To validate the RNA-seq results, samples from the same treatment and RNA extraction for RNA-seq were also used for cDNA synthesis. The RNA was reverse-transcribed to cDNA using the PrimeScript RT Reagent Kit with gDNA Eraser (Takara, Shiga, Japan). Twenty DEGs were verified by SYBR^®^ Premix Ex Taq^™^ (Takara) using a Bio-Rad CFX96 Real-Time PCR System (Bio-Rad Laboratories, Hercules, CA, United States). The β-actin gene was used as the internal control gene. All primers used are listed in Supplementary Table S3. The relative expression level of each targeted gene was calculated using the 2^−△△Ct^ method. Three replicates of each qRT-PCR analysis were performed for each gene.

### Statistical analysis

All tests were performed in triplicate. All the statistical analyses were performed with GraphPad Prism v7.0 (GraphPad Software, San Diego, CA, United States). The significance of differences between the means of the treatments was analyzed with one-way ANOVA followed by paired-samples t tests at the 5% significance level (*p* < 0.05).

## Results

### Isolation of fungi and pathogenicity test

**Figure 1:**
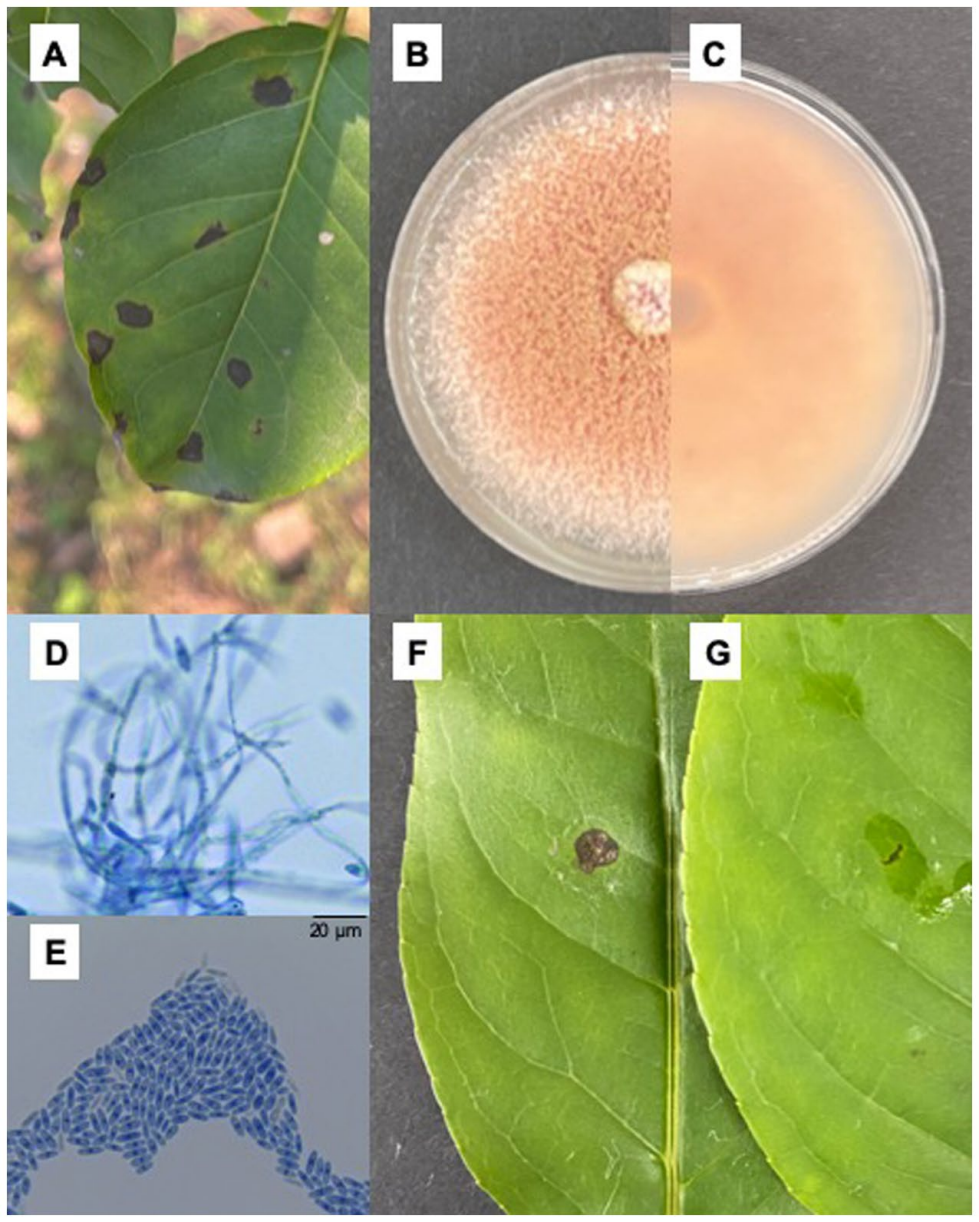
Isolation of fungi and pathogenicity test. **(A)** Brown sunken necrotic leaf spots causing by *Colletotrichum fioriniae* on *Ilex macrocarpa* in the field; **(B,C)** Colonial morphology of XLHR17 on PDA; **(D,E)** Mycelium and conidiospore of XLHR17; **(F,G)** Symptoms on leaves 7 days after inoculated by XLHR17 and sterile distilled water.

In an artificial forest in Nanchong, Sichuan, China (EN), *I. macrocarpa* trees exhibited leaf spot disease from April to September in 2021 and 2022. The symptoms usually start at the leaf margin or tip with brown necrotic spots in the center, which gradually expand and merge to form a large irregular necrotic area with gray spots in the center surrounded by a dark brown margin, resulting in inward curling of the leaves and defoliation (Figures 1A–E). The morphology of 19 out of 26 isolates from diseased leaves of *I. macrocarpa* was similar to that of *C. fioriniae* described by Talhinhas and Baroncelli (2021). The colonies were initially white and light gray and then gradually became light pink in the center, finally forming orange conidial masses. The reverse side of the colony turned from pink to red (Figures 1B,C). The conidia were hyaline, smooth-walled, slender, and rounded with tapered ends and measured 3.6–6.6 × 8.5–17.8 μm in size (*n* = 50) (Figures 1D,E). All the isolates were pathogenic to the detached *I. macrocarpa* leaves, and XLHR17 was the most strongly pathogenic strain. After 7 days, the inoculated leaves showed brown to black spots, while the control remained asymptomatic. The morphology of the fungal colonies reisolated from the symptomatic leaves was the same as that of the colonies produced by the original isolate used for inoculation, which conforms to Koch’s postulate (Figures 1F,G).

### Phylogenetic analyses

**Figure 2:**
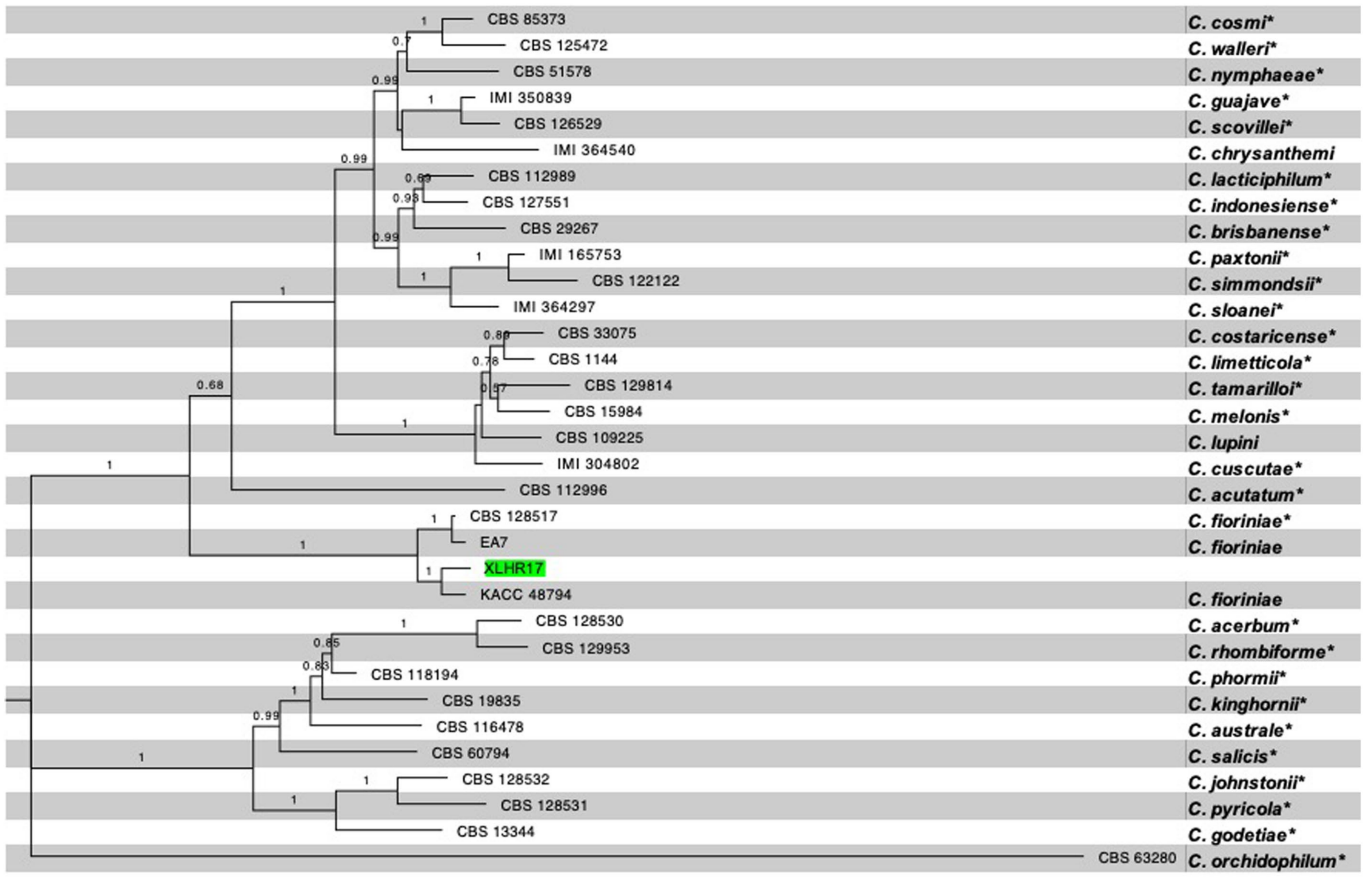
Phylogenetic tree generated by maximum likelihood analysis based on concatenated sequences of the *ITS*, *HIS3*, *CHS-1*, *ACT*, *TUB2*, and *GAPDH*. The dataset contained 31 ingroup taxa, and the tree is rooted with *C. orchidophilum*. The ex-type (exepitype) strains are marked with a *. RA × ML bootstrap values equal to or greater than 70% are shown at the nodes. The isolate used in present study, XLHR17, is highlighted in green.

A multilocus approach was used to confirm pathogen identity. The internal ITS, *HIS3*, *CHS-1*, *ACT*, *TUB2*, and *GAPDH* regions of the representative isolate XLHR17 were amplified and sequenced. The pairwise alignments of the ITS, HIS3, CHS-1, ACT, *TUB2*, and GAPDH sequences were 99% identical to those of *C. fioriniae*, with the GenBank accession numbers MN842794 (567/569 bp), MZ557420 (368/367 bp), MT954330 (260/261 bp), LC381035 (237/239 bp), MK967342 (727/727 bp), and MZ229316 (237/237 bp), respectively. The resulting sequences were deposited in GenBank (accession nos: ITS: ON714636; HIS3: OQ025283; CHS-1: OQ025281; ACT: OQ025278; *TUB2*: OQ025282; and GAPDH: OQ025279). A phylogenetic tree was constructed using concatenated sequences (maximum likelihood method) with MEGA 11 (1,000 bootstrap replications) to further reveal interspecies relationships. Phylogenetic analysis classified XLHR17 into the *C. fioriniae* clade and showed that it shared the closest phylogenetic relationship with *C. fioriniae* (KACC48794) (Figure 2). Through morphological and molecular analyses, the fungus that caused brown spots on *I. macrocarpa* leaves was confirmed to be *C. fioriniae*.

### Effect of chemical fungicides at different concentrations on the mycelial growth of *Colletotrichum fioriniae*

**Figure 3:**
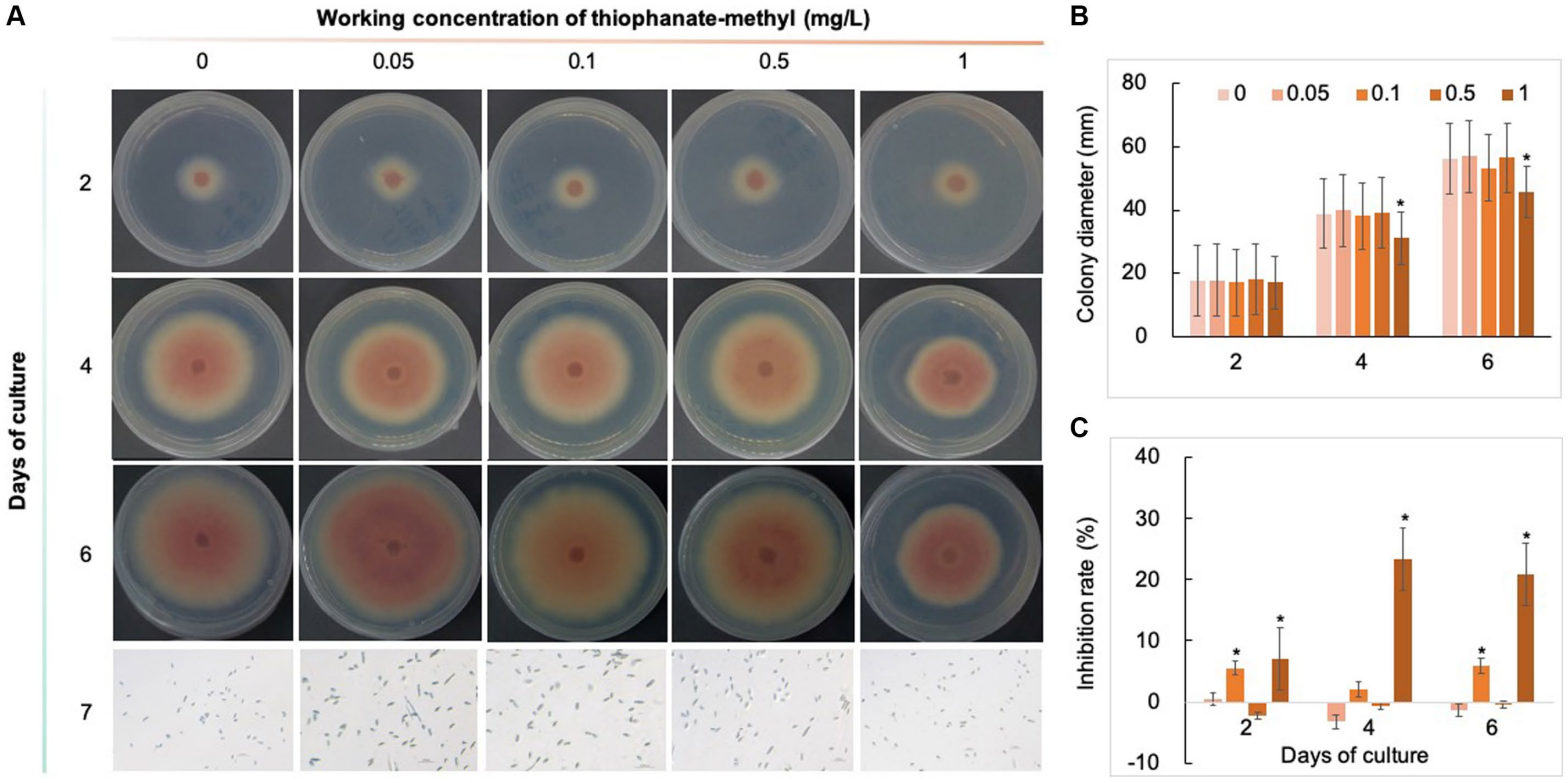
Inhibitory effect of thiophanate-methyl on *C. fioriniae*. **(A)** The appearance of colony size and spore number of *C. fioriniae* inhibited by thiophanate-methyl; **(B)** Colony diameter; **(C)** Inhibition rate. *p* < 0.05 was marked with a *. Similarly hereinafter.

**Figure 4:**
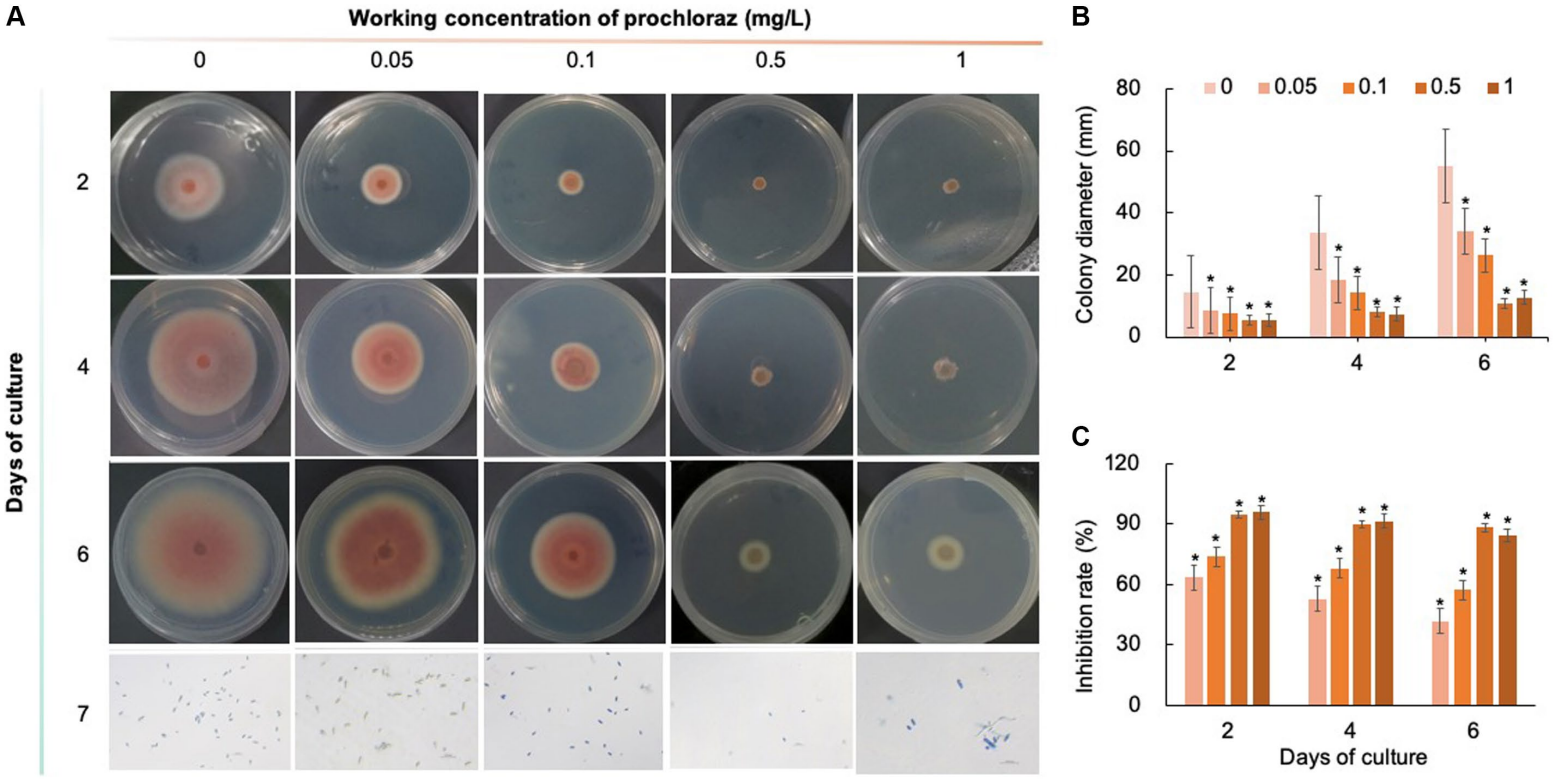
Inhibitory effect of prochloraz on *C. fioriniae*. **(A)** The appearance of colony size and spore number of *C. fioriniae* inhibited by t prochloraz; **(B)** Colony diameter; **(C)** Inhibition rate.

**Figure 5:**
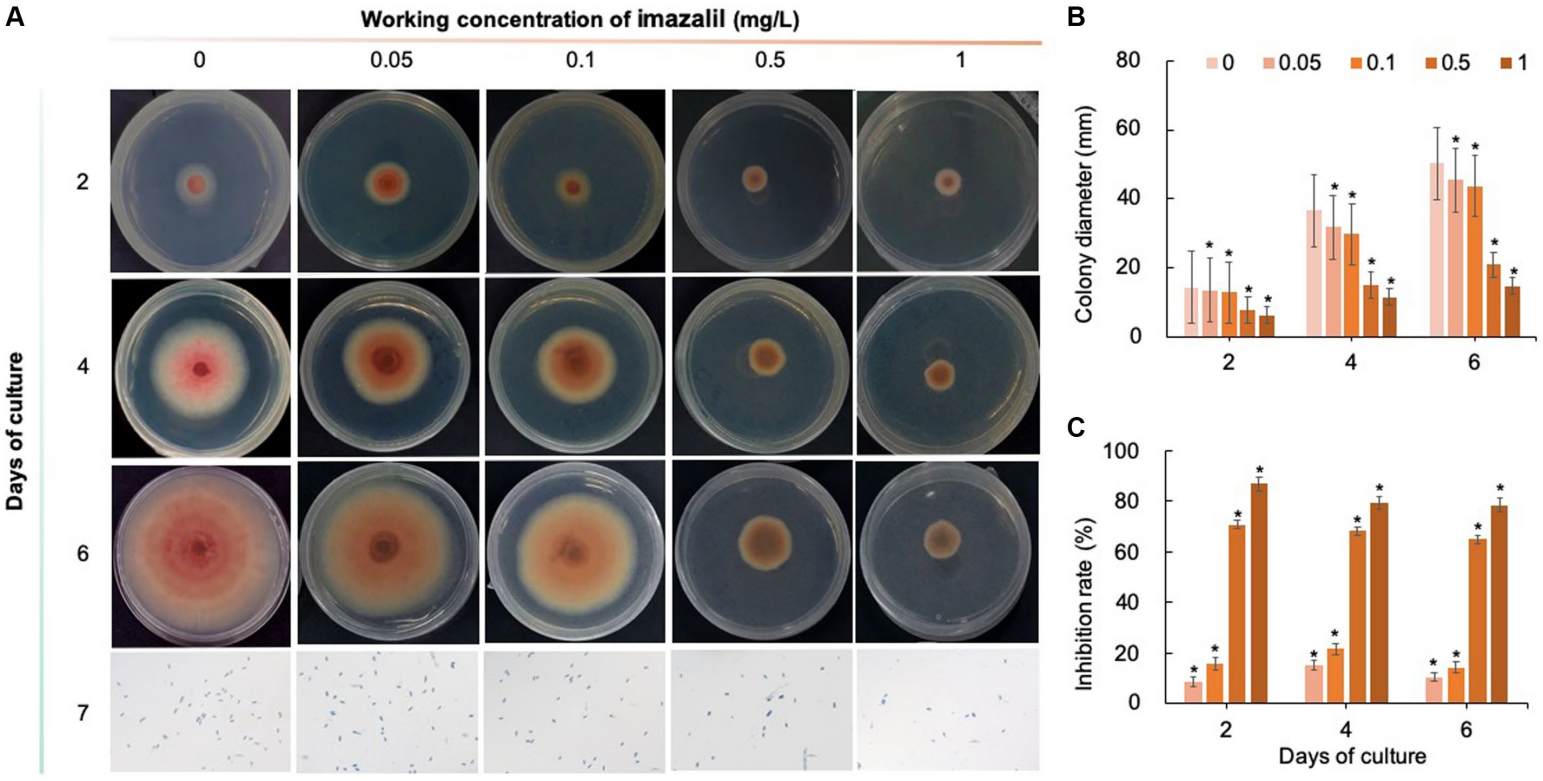
Inhibitory effect of imazalil on *C. fioriniae*. **(A)** The appearance of colony size and spore number of *C. fioriniae* inhibited by imazalil; **(B)** Colony diameter; **(C)** Inhibition rate.

**Figure 6:**
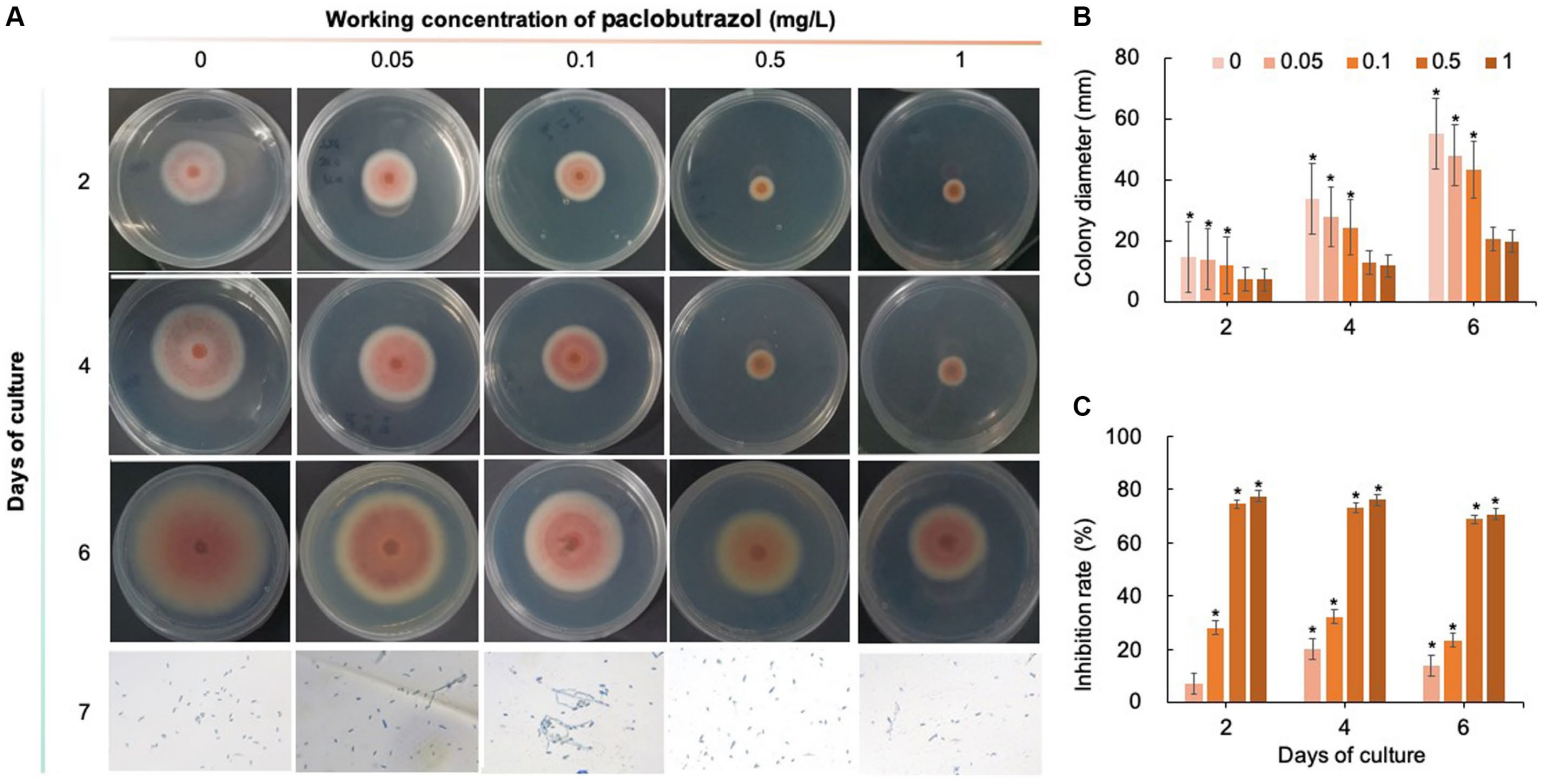
Inhibitory effect of paclobutrazol on *C. fioriniae*. **(A)** The appearance of colony size and spore number of *C. fioriniae* inhibited by paclobutrazol; **(B)** Colony diameter; **(C)** Inhibition rate.

**Figure 7:**
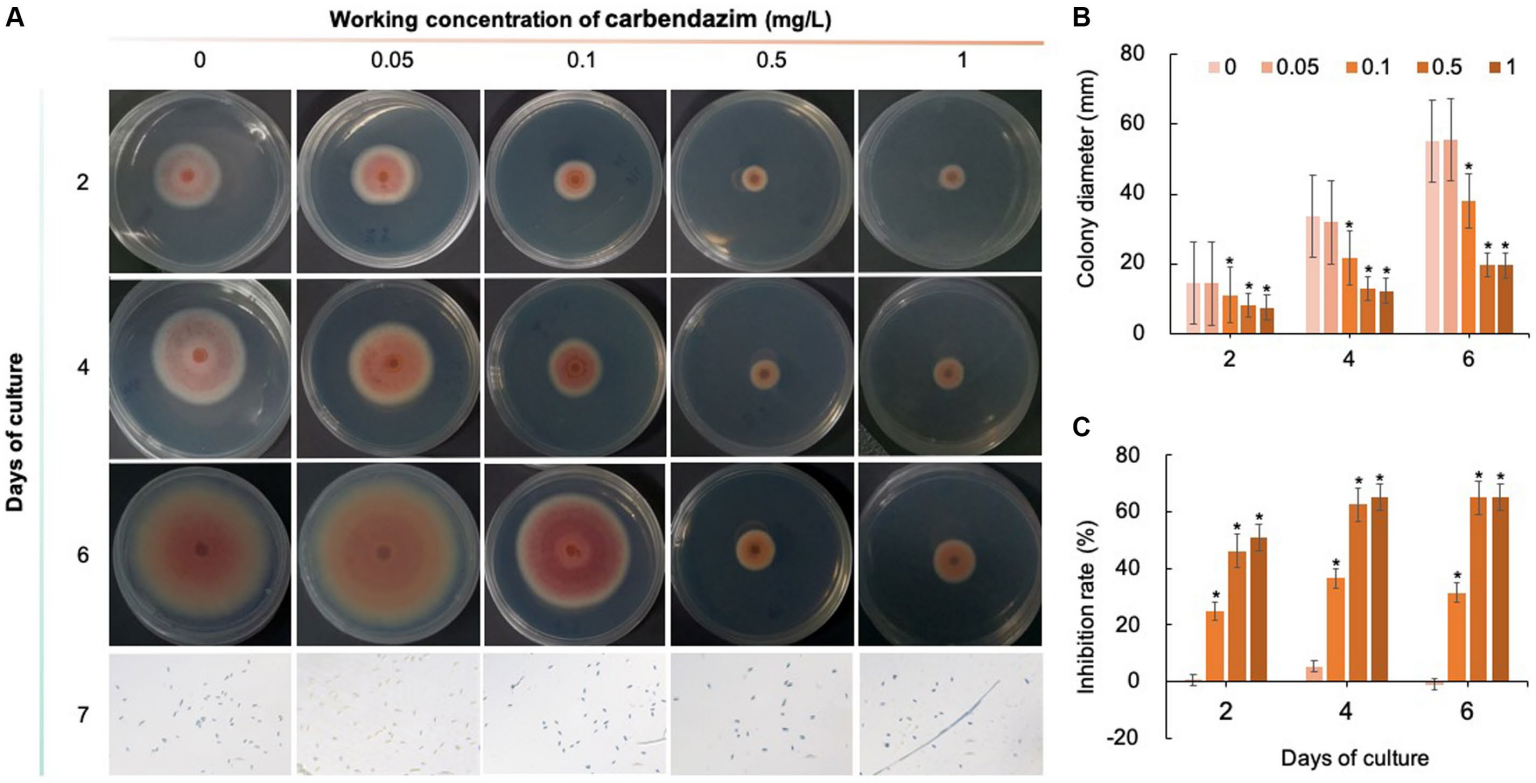
Inhibitory effect of carbendazim on *C. fioriniae*. **(A)** The appearance of colony size and spore number of *C. fioriniae* inhibited by carbendazim; **(B)** Colony diameter; **(C)** Inhibition rate.

Five chemical fungicides and three biological fungicides were used to investigate their inhibitory effects on *C. fioriniae*. The colony size of the CK group did not increase after 6 days of culture, so the colony size on the sixth day was recorded to analyze the inhibitory effects (Figures 3–7). The results revealed that the five chemical fungicides inhibited *C. fioriniae* to different degrees. Among them, thiophanate-methyl had the weakest inhibitory effect, showing only slight inhibitory effects at a concentration of 1 mg/L and an inhibition rate of 25.56% (Figure 3). The other four chemical fungicides had relatively significant fungistatic effects even at low concentrations (0.05 mg/L), and the effects became stronger with increasing mass concentration. For example, when the mass concentration of prochloraz was only 0.05 mg/L, its inhibition rate reached 35.85%, and there were no significant differences between the effects of 0.5 mg/L and 1 mg/L prochloraz; the inhibition rates were 81.67 and 77.20%, respectively (Figure 4). Imazalil and paclobutrazol both showed no significant difference compared to CK at concentrations of 0.05 mg/L and 0.1 mg/L but had significantly greater effects at concentrations of 0.5 mg/L and 1 mg/L, with both showing the best inhibitory effect at 1 mg/L, with inhibition rates of 61.40 and 70.67%, respectively (Figures 5, 6). However, the different concentrations of carbendazim all had significantly stronger inhibitory effects than CK, except at a concentration of 0.05 mg/L, and there were no differences between the effects of 0.5 mg/L and 1 mg/L carbendazim (the inhibition rates were 64.02%), while the 0.1 mg/L had a weaker inhibitory effect (with an inhibition rate of 29.10%) (Figure 7). Thus, based on the comprehensive evaluation of the inhibitory effects of the five chemical agents against *C. fioriniae*, the strengths of the effects were in the following order: prochloraz > imazalil > carbendazim > paclobutrazol > thiophanate-methyl.

### Effect of biological fungicides at different concentrations on the mycelial growth of *Colletotrichum fioriniae*

**Figure 8:**
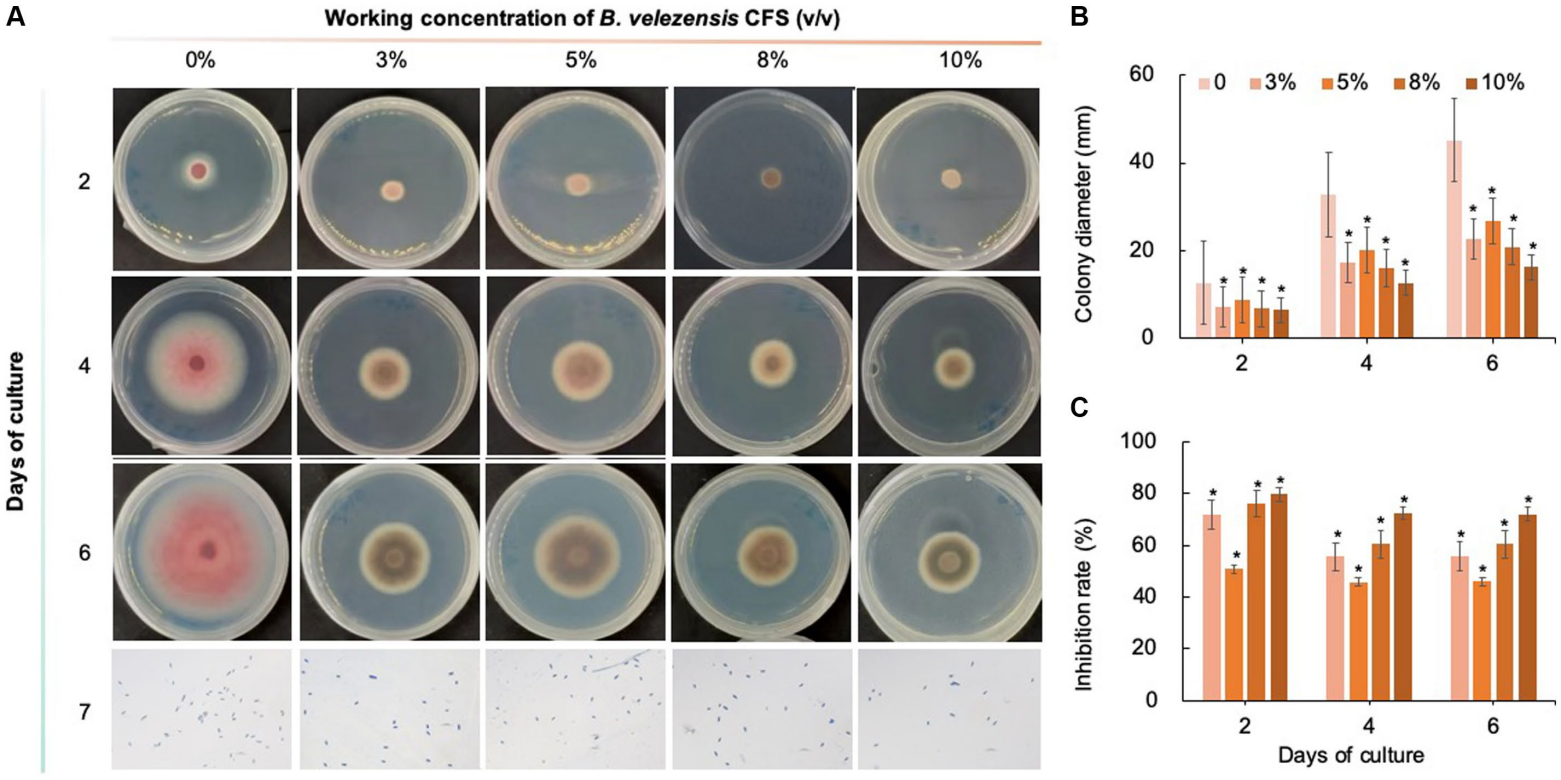
Inhibitory effect of *B. velezensis* CFS on *C. fioriniae*. **(A)** The appearance of colony size and spore number of *C. fioriniae* inhibited by *B. velezensis* CFS; **(B)** Colony diameter; **(C)** Inhibition rate.

**Figure 9:**
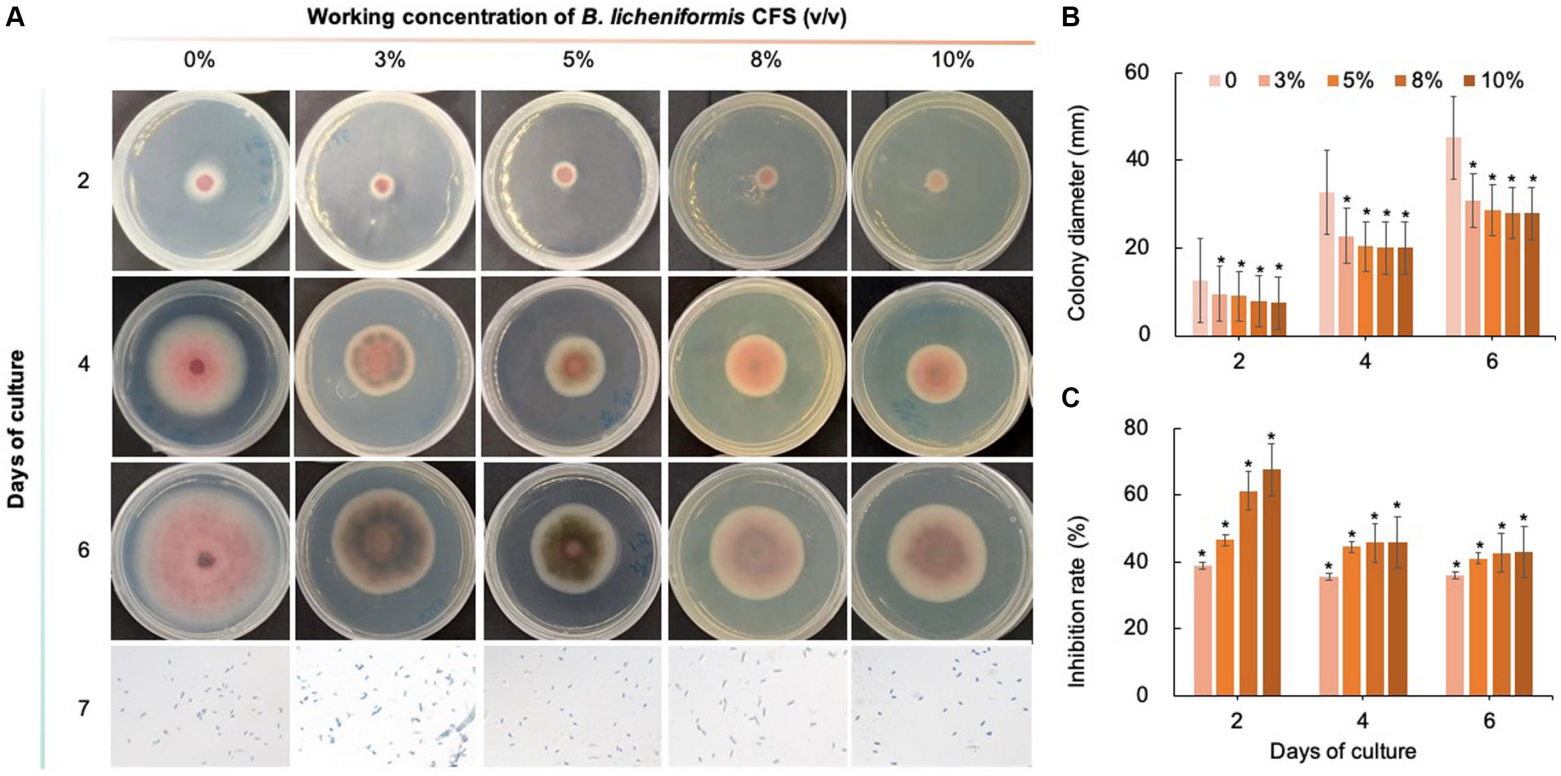
Inhibitory effect of *B. licheniformis* CFS on *C. fioriniae*. **(A)**. The appearance of colony size and spore number of *C. fioriniae* inhibited by *B. licheniformis* CFS; **(B)** Colony diameter; **(C)** Inhibition rate.

**Figure 10:**
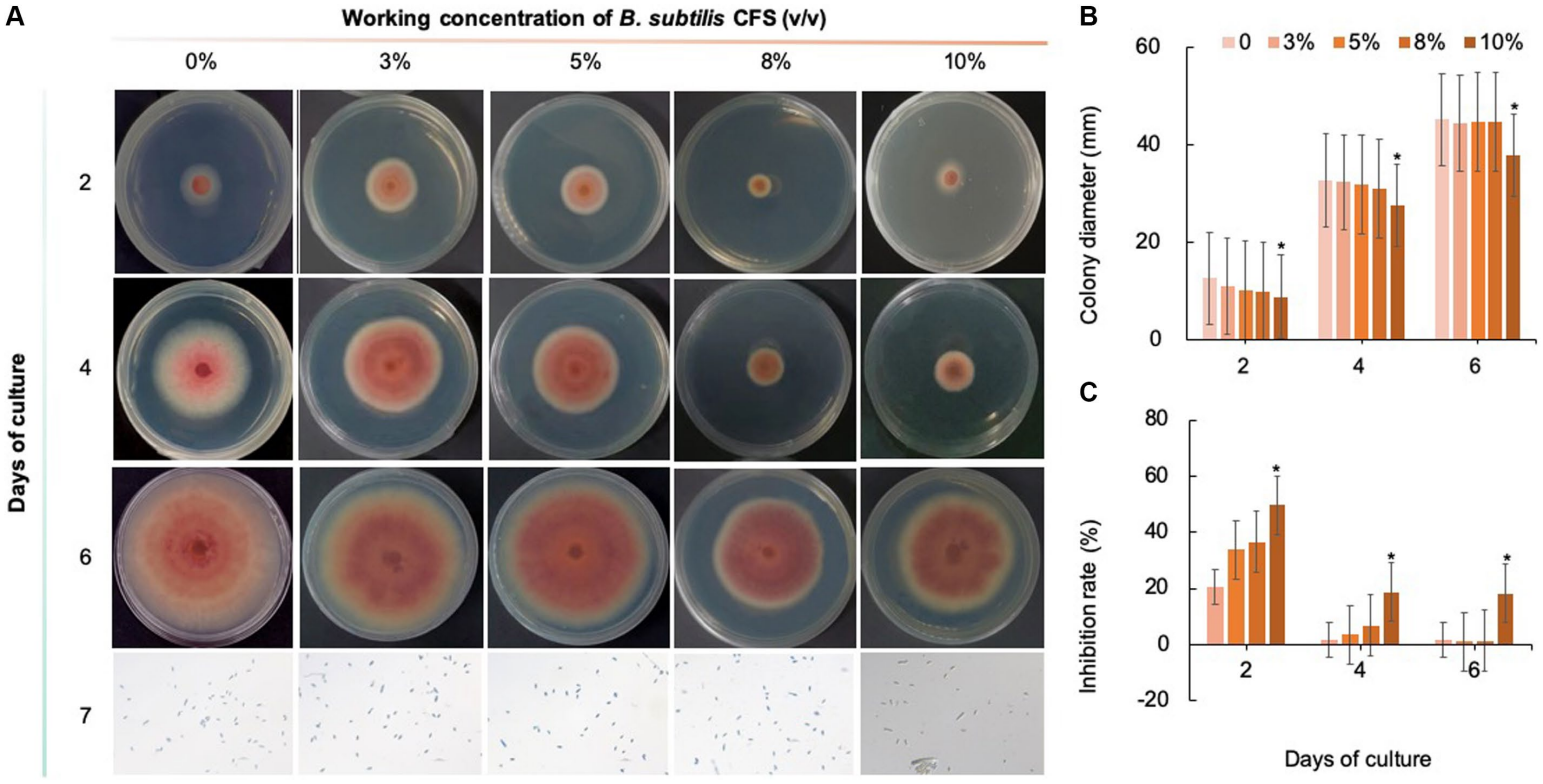
Inhibitory effect of *B. subtilis* CFS on *C. fioriniae*. **(A)** The appearance of colony size and spore number of *C. fioriniae* inhibited by *B. subtilis* CFS; **(B)** Colony diameter; **(C)** Inhibition rate.

As shown in Figures 8–10, compared with that on the control medium, the colony diameter of *C. fioriniae* on PDA medium supplemented with biological fungicides decreased. There was a positive correlation between the concentration of biological fungicides and growth inhibition. Application of the CFS of *B. velezensis* at 5% resulted in an approximately 48.85% inhibition rate, and when the concentration reached 10%, the inhibition rate reached 59.42% (Figure 8). *B. licheniformis* also had negative effects on the colony diameter of *C. fioriniae* in a concentration-independent manner. Treatment with 3, 5, 8, and 10% *B. licheniformis* CFS resulted in 39.81, 44.81, 45.77, and 46.73% inhibition, respectively (Figure 9). However, the effect of the application of *B. subtilis* CFS was not significantly different from that of the control (Figure 10). Similarly, the spore number of *C. fioriniae* was also inhibited by biofungicides. The effects of the three biofungicides against *C. fioriniae* were comprehensively evaluated and ranked as follows: *B. velezensis* > *B. licheniformis* > *B. subtilis*.

### Transcriptome analysis of *Colletotrichum fioriniae* after *Bacillus velezensis* CFS treatment

To further elucidate the inhibitory mechanisms of *B. velezensis* CFS, the transcriptional profile of *C. fioriniae* was determined after 10% *B. velezensis* CFS treatment. After filtering the adaptors and low-quality sequences, an average of 4.76 Gb of clean data for each sample was produced. Of these, 95.4% of the clean reads were aligned to the *C. fioriniae* reference genome for gene expression analysis (Supplementary Table S4). In total, 5,260 (4,440) DEGs with at least a 1.0 (1.5)-fold change in expression were identified, including 1,446 (1,050) upregulated genes and 3,814 (3,390) downregulated genes after *B. velezensis* CFS treatment (Supplementary Figure S1).

**Figure 11:**
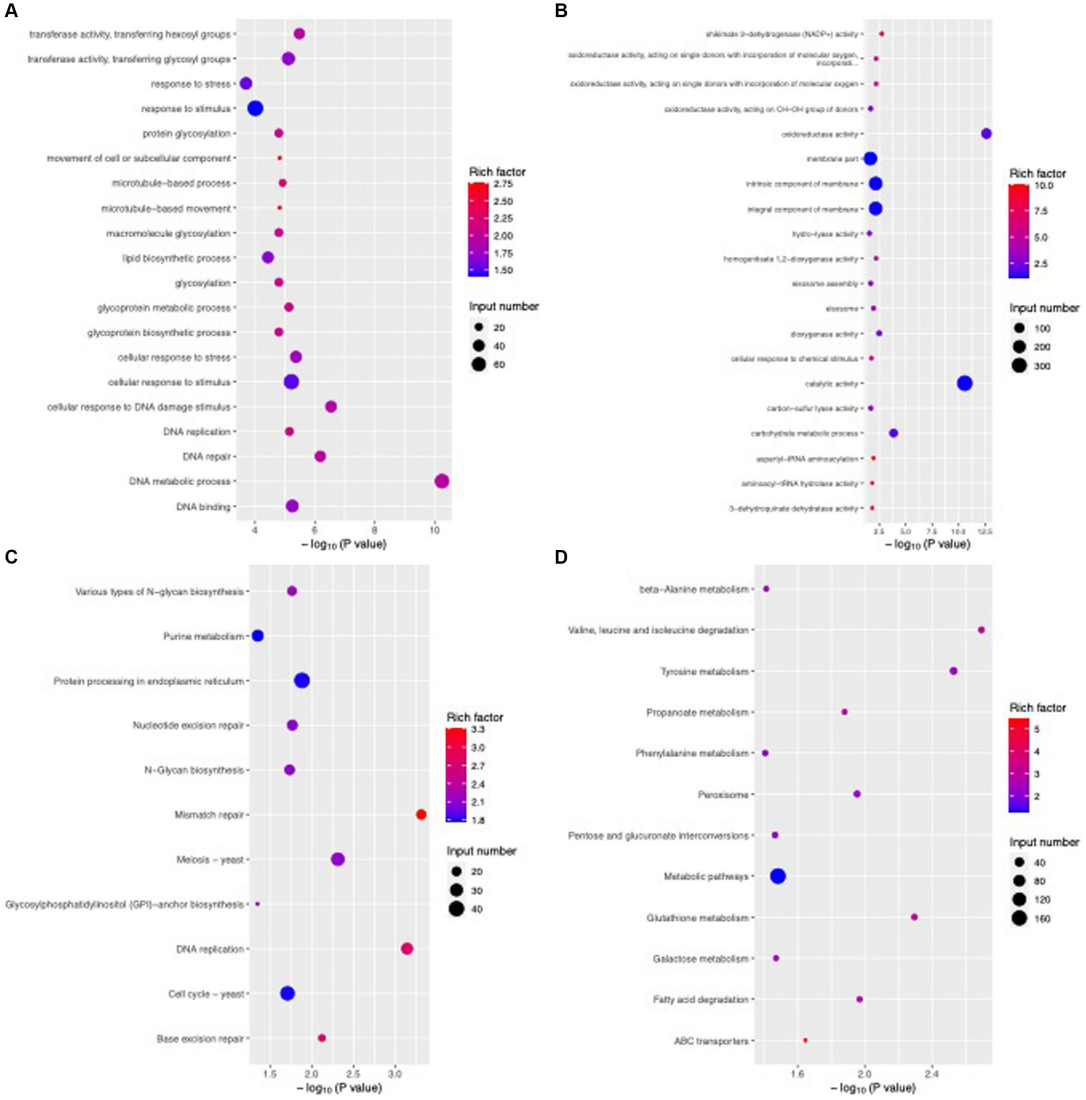
Enriched GO terms and KEGG pathways of DEGs after *B. velezensis* CFS treatment. GO enrichment analysis of upregulated genes **(A)** and downregulated genes **(B)**. KEGG enrichment analysis of upregulated genes **(C)** and downregulated genes **(D)**. The size of the dots denotes the number of enriched genes, and the color intensity denotes the significant level.

To reveal the functions and regulatory pathways of these DEGs, we performed GO and KEGG pathway enrichment analyses. Through the GO function enrichment analysis, the upregulated DEGs were found to be involved mainly in catalytic activity, membrane part, integral component of membrane, intrinsic component of membrane, oxidoreductase activity and carbohydrate metabolic process, while the downregulated DEGs participated mainly in cellular process, macromolecule metabolic process, cellular macromolecule metabolic process, heterocycle metabolic process, nucleobase-containing compound metabolic process and nucleic acid metabolic process (Figures 11A,B). KEGG pathway analysis indicated that the upregulated DEGs were mainly enriched in metabolic pathways; tyrosine metabolism; peroxisome, valine, leucine and isoleucine degradation; pentose and glucuronate interconversions; glutathione metabolism; and phenylalanine metabolism (Figure 11C). The downregulated DEGs were mainly enriched in the pathways of protein processing in the endoplasmic reticulum, the cell cycle, meiosis, purine metabolism, DNA replication, nucleotide excision repair and N-glycan biosynthesis (Figure 11D). These results demonstrated that *B. velezensis* CFS treatment might inhibit the growth of *C. fioriniae* by interfering with the structure and function of ribosomal and genetic information processing, such as DNA replication and translation.

### DEGs involved in basic metabolic pathways

**Figure 12:**
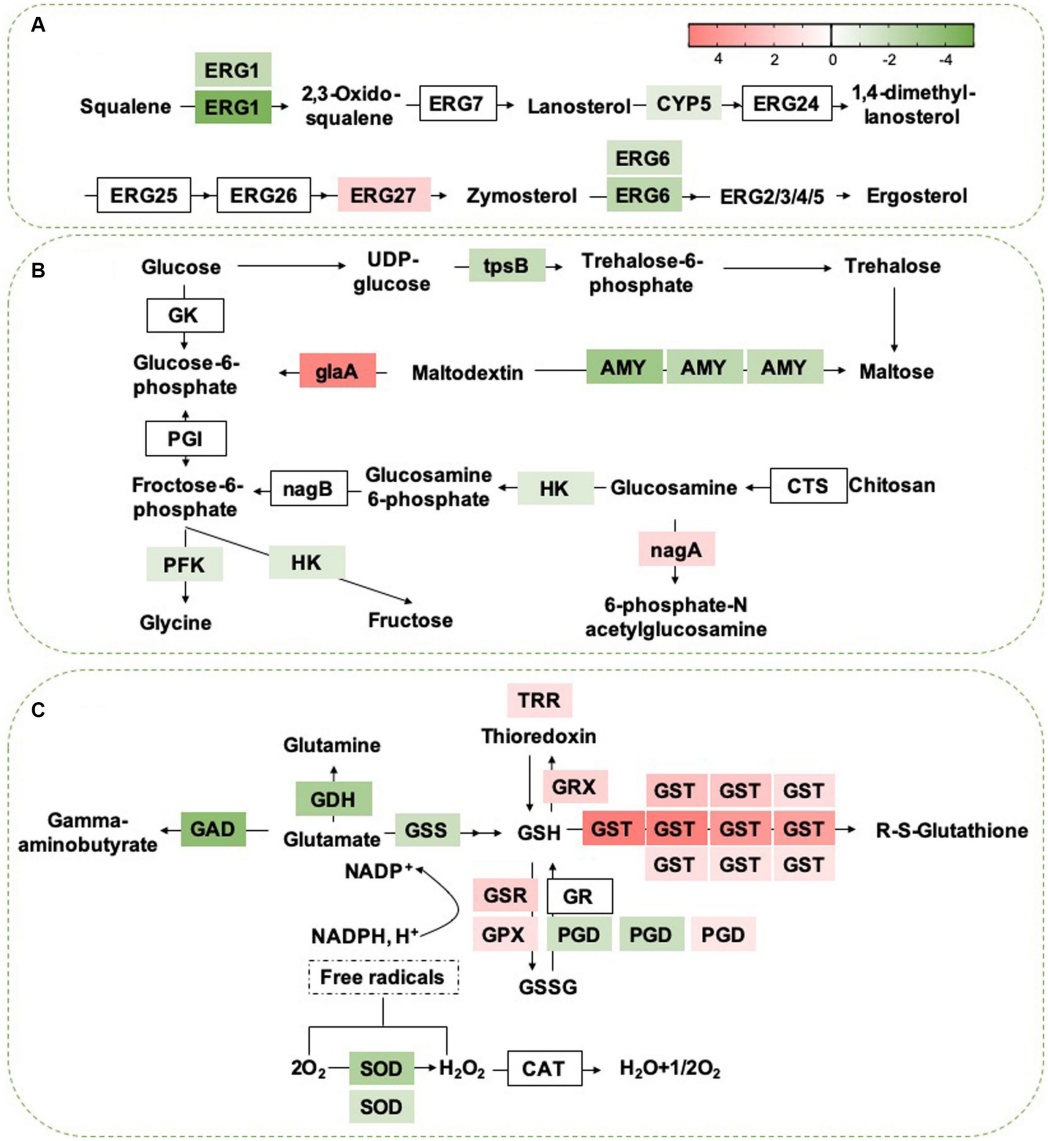
**(A)** Heat map of DEGs that involved in Steroid biosynthesis. ERG1, squalene epoxidase; ERG7, lanosterol synthase; CYP5, cytochrome b5; ERG24, C-14 sterol reductase; ERG25, C-4 sterol methyloxidase; ERG26, C-4 sterol decarboxylase; ERG27, C-3 sterol Keto reducatse; ERG6, C-24 Sterol methyl-transferase; ERG2, C-8 Sterol isomerase; ERG 3, C-5 Sterol desaturase; ERG 5:C-22 Sterol desaturase; ERG 4, C-24 sterol reductase. The heat map was generated by log (fold change) using Graphpad prism software: red, increase; green, decrease. **(B)** Heat map of DEGs that may be involved in sugar metabolism. GK, Glucokinase; PGI, Phosphoglucose isomerase; HK, Hexokinase; tpsB, trehalose 6-phosphate synthase; AMY, Alpha-amylase A; gLaA, Glucoamylase; CTS, Chitosanase; nagA, 6-Phosphate-N-acetylglucosamine deacetylation; nagB, 6-phosphate glucosamine deaminase; PFK, phosphofructokinase. The heat map was generated by log (fold change) using Graphpad prism software: red, increase; green, decrease. **(C)** Heat map of DEGs that may be involved in sugar metabolism. GDH, Glutamate dehydrogenase; argJ, Amino-acid acetyltransferase; GSS, glutathione synthase; GSH, glutathione; GR, glutathione reductase; PGD:6-phosphogluconate dehydrogenase; GPX, glutathione peroxidase; GTT, Glutathione transporter; GSSG, glutathione disulfide; GST, Glutathione S-transferase; TRR, Thioredoxin reductase; GRX, Glutaredoxin; SOD, Superoxide dismutase; CAT, Catalase. The expression image was generated using Graphpad prism software: red, increase; green, decrease.

Based on the GO and KEGG analyses, we selected some critical pathways that may be associated with the antifungal activity of *B. velezensis* for further analysis. For example, the results showed that the expression of multiple DEGs related to glycerophospho lipid and ether lipid metabolism pathways was activated by *B. velezensis* CFS treatment, particularly 4 genes encoding phospholipase C (PLC, 2 genes upregulated and 2 genes downregulated), the key enzymes that catalyze the decomposition of phospholipids. Moreover, *B. velezensis* CFS treatment altered the transcription of 8 genes in the steroid biosynthetic pathway; except for the up regulated gene ERG27, all of these genes were down regulated in the treatment group (Figure 12A). In addition, many genes involved in the oxidation–reduction process, including 24 genes related to alcohol dehydrogenase (16 genes up regulated and 8 genes down regulated), 5 genes encoding acyl-CoA dehydrogenase (all up regulated), and 12 genes in the acetyltransferase (GNAT) family (8 genes up regulated and 4 genes down regulated), were differentially expressed after *B. velezensis* CFS treatment (Supplementary Table S5). Additionally, the expression of 6 genes related to sugar metabolism (encoding phosphofructokinase, alpha amylase, trehalose-phosphatase, and hexokinase) was down regulated, and the expression of 2 genes (encoding glucoamylase and N-acetylglucosamine-6-phosphate deacetylase) was induced by *B. velezensis* CFS (Figure 12B). These findings suggest that *B. velezensis* CFS disrupted basic metabolic pathways in *C. fioriniae*, including glucose metabolism, lipid metabolism, and other carbon metabolism. Moreover, *B. velezensis* CFS could also influence the pathogenicity of *C. fioriniae* by reducing the expression of plant-pathogen interaction genes (GTPase, glycerol kinase, and GAPR1); the expression of genes encoding the autophagy proteins ATG8 and ATG9 was induced, and ATG17 was down regulated. Therefore, it would be interesting to further investigate whether autophagy is related to hyphal swelling and deformation induced by *B. velezensis* CFS treatment.

### DEGs in the glutathione metabolism pathway

Considering that *B. velezensis* CFS treatment disrupted multiple metabolic pathways in *C. fioriniae*, we analyzed the top five enriched pathways and found that the expression of 26 genes related to membranes (including the extracellular membrane, mitochondrial inner membrane and transmembrane protein) (Supplementary Table S6) and 23 genes related to ribosome metabolism decreased markedly after *B. velezensis* CFS treatment (Supplementary Table S7), which suggested that *B. velezensis* CFS treatment interfered with the growth of *C. fioriniae* mainly by damaging cellular ribosomes, genetic information processing and cell membranes. To reduce the stress of *B. velezensis* CFS and for survival in unfavorable environments, we found that the glutathione metabolism pathway was also significantly enriched, and proteins encoded by the corresponding genes glutathione S-transferase (GST, 10 DEGs), Glutathione peroxidase (GPX), glutathione-disulfide reductase (GSR), glutaredoxin (Grx) and thioredoxin reductase (TRR) were upregulated by *B. velezensis* CFS treatment (Figure 12C). Moreover, SD treatment dramatically increased the expression of nine ABC and seventeen MFS protein genes, while the expression of three ABC and seventeen MFS protein genes decreased (Supplementary Tables S8, S9). These results suggested that *B. velezensis* CFS treatment also stimulated several detoxification-related pathways in *C. fioriniae*, such as glutathione metabolism and pathways involving the ABC and MFS transporter protein families, to reduce the damage caused by *B. velezensis* CFS and promote the formation of complex interactions between *C. fioriniae* and *B. velezensis* CFS.

### Validation of RNA-seq results by qRT-PCR analysis

DEGs selected for validation were key genes or genes with significant differences in expression in pathways of interest. The results showed that all 23 selected DEGs exhibited almost the same expression pattern, except for one gene (COL516b_008851), which was slightly upregulated in the treatment group according to the RNA-seq analysis but showed no significant differences according to the qRT-PCR analysis (Supplementary Figure S2). These results verified the overall reliability of the RNA-seq data.

## Discussion

*Colletotrichum* spp. are fungal pathogens distributed worldwide that cause anthrax in plants. This disease can result in significant reductions in plant yield and quality. Currently, chemical control is the primary method used to inhibit pathogenic *Colletotrichum* spp. However, its application is limited due to the diversity of *Colletotrichum* spp. species and pesticide residues (Bordoh et al., 2020). In addition to breeding disease-resistant varieties, another effective approach is to select the type and dosage of fungicides based on the specific anthrax species (Dowling et al., 2020). In this study, we used morphological and phylogenetic tree analyses to identify and report that *C. fioriniae* is the pathogen responsible for anthrax in *I. macrocarpa*. With this information, we investigated the activities of five chemical fungicides against *C. fioriniae* on *I. macrocarpa*. Our findings showed that prochloraz exhibited the highest activity against *C. fioriniae*. A low concentration (0.1 mg/L) of prochloraz achieved a 50% inhibition rate, indicating its effectiveness at this concentration. Prochloraz functions as a broad-spectrum fungicide by inhibiting sterol biosynthesis and interfering with cell wall biosynthesis (Zhang et al., 2021; Yang et al., 2022), which was confirmed in our study.

However, biological control is preferred because it is environmentally friendly and less likely to cause mutations in pathogens. *Bacillus* spp. are known to produce secondary metabolites that can be used as drugs or pesticides, or components that can restructure the soil microbiome structure and function (Zhang et al., 2020; Salazar et al., 2023; Sarangi and Ramakrishnan, 2023). In this study, three biological fungicides, namely *B. velezensis*, *B. licheniformis*, and *B. subtilis*, were examined for their inhibitory effects on *C. fioriniae* isolated from *I. macrocarpa*. The results demonstrated that *B. velezensis* and *B. licheniformis* had some inhibitory effect on *C. fioriniae*, but *B. velezensis* showed a significant advantage over the other two biofungicides in terms of its inhibitory effects, with an IC50 of 3% CFS. This suggests that different biological fungicides have varying degrees of control, possibly due to differences in their mechanisms of action. However, the antifungal mechanism of *B. velezensis*, particularly at the molecular level, still requires identification and understanding, which could greatly facilitate its use in plant disease control. Additionally, this study revealed that the *B. velezensis* CFS disrupted the mycelial morphology of *C. fioriniae*, although the underlying mechanism remains unclear.

Transcriptome analysis of *C. fioriniae* treated with *B. velezensis* CFS was used to investigate the inhibitory mechanism of *B. velezensis*. The results showed that *B. velezensis* CFS reduced the expression of multiple genes in the ribosome pathway, indicating that interference with ribosome structure and function might be an intrinsic reason for the inhibitory effect of the *B. velezensis* CFS on *C. fioriniae*. Ribosomes are mainly composed of small ribosomal subunits and large subunits, which are responsible for “reading” RNA information and for the formation of polypeptide chains, respectively. As an important site for the processing of genetic information, ribosomes participate directly in the regulation of cellular protein synthesis and control life activities indirectly, and any changes leading to ribosomal component alterations can directly affect the normal function of ribosomes; thus, ribosomes are a common target of antifungal agents (Hurtado-Rios et al., 2022; Ivanov et al., 2022). RNA-seq analysis of *P. digitatum*, *B. cinerea*, and *Aspergillus flavus* revealed that the application of antifungal compounds disrupted the expression of genes related to ribosome biogenesis and protein biosynthesis and impeded protein synthesis (Roca-Couso et al., 2021; Dimkić et al., 2022; Lei et al., 2023), which is consistent with our results. In addition, the expression of one gene encoding aminoacyl-tRNA synthetase, which catalyzes the binding of transfer (t)RNA to its cognate amino acids to form aminoacyl-tRNA for synthesizing proteins at specific positions of the ribosome, is reduced by *B. velezensis* CFS treatment. The expression of genes related to transcription and DNA replication was also disrupted, and these changes might be due to ribosome damage caused by *B. velezensis* CFS treatment. Consistent with this, we found that the expression of most of the genes related to ribosome composition (40S ribosomal proteins RPS5, RPS8, RPS10, RPS15, RPS27/33, and RPS54 and 60S ribosomal proteins RPL1, RPL3, RPL7/12, RPL11, RPL14, RPL16, RPL17, RPL22, RPL25, RPL31, and RPL36) decreased. These results also confirmed that ribosomes might be direct targets of *B. velezensis* CFS and that the initial destruction of ribosome components interferes with genetic information processing and leads to the inhibition of *C. fioriniae*. Moreover, two genes encoding LETM-1 ribosome binding proteins, which regulate mitochondrial integrity, the production of endogenous reactive oxygen species and mycotoxin biosynthesis (Natarajan et al., 2021), were downregulated by the *B. velezensis* CFS, further suggesting potential target genes for *B. velezensis* CFS.

According to the DEG analysis, cell membranes were also possible targets of *B. velezensis* CFS. The destruction of cell membranes can disrupt cellular components, disrupt cell homeostasis and lead to cell death. In the present study, we found that various genes involved in cell membrane lipid metabolism, particularly sphingolipid metabolism, were obviously downregulated by *B. velezensis* CFS treatment. Moreover, *B. velezensis* CFS treatment also affected the expression of PLC, thereby disrupting the synthesis and degradation of phospholipids (Adigun et al., 2021; Yan et al., 2021), which can damage the integrity of cell membranes. In addition, sugar metabolism in *C. fioriniae* was disrupted by *B. velezensis* CFS treatment, and the expression of multiple genes related to alternative carbon metabolism was also affected. In our previous study, *B. velezensis* CFS treatment might have led to an energy shortage in *P. olsonii*, which accelerated alternative carbon metabolism, and the decreased energy status further disturbed cell homeostasis (Zhang et al., 2023). Similarly, other antifungal agents have also been reported to affect energy metabolism and reduce the pathogenicity of pathogens (Chen et al., 2020; Owens and Doyle, 2021; Liu et al., 2022; Wijnants et al., 2022). Thus, damage to the cell membrane system and disturbance of energy metabolism might ultimately accelerate cell death, which partly explains the antifungal effects of *B. velezensis* CFS treatment. Moreover, autophagy is associated with multiple physiological and pathological functions in filamentous fungi and is possibly a target of several antifungal agents (Lai et al., 2022; Slavin and Bach, 2022). We found that the expression of ATG8 and ATG9 was induced by *B. velezensis* CFS, which indicated that autophagy in *C. fioriniae* might be a target of *B. velezensis*.

Glutathione metabolism plays a key role in various processes, including amino acid transport, enzyme activity regulation, tissue redox homeostasis maintenance, and detoxification of harmful substances. These processes collectively contribute to the protection of fungi against unfavorable environments and external treatments (Mattila et al., 2022; Wangsanut and Pongpom, 2022; Yaakoub et al., 2022). Our study revealed that the expression of multiple genes associated with glutathione metabolism (GST, GRX, TRR, GSR, GPX) was significantly increased following treatment with *B. velezensis* CFS. This suggests that *C. fioriniae* may reduce the toxicity of *B. velezensis* CFS by increasing the glutathione content in cells. Notably, MBL and GST are also implicated in pathogen virulence (Yahiaoui et al., 2021; Hu J. et al., 2022), further highlighting the potential of *B. velezensis* CFS in controlling anthracnose in plants. Moreover, *B. velezensis* also induces the expression of ABC and MFS transporters, which may help reduce the toxicity of its CFS by facilitating the removal of harmful substances from cells through ATP hydrolysis and substrate transfer across the plasma membrane (Pontes et al., 2020; Broberg et al., 2021). However, further research is needed to determine how *C. fioriniae* mitigates the toxic effects of *B. velezensis* CFS on itself.

## Conclusion

The present study is the first report of *C. fioriniae* causing anthracnose on *I. macrocarpa* in China. This finding provides a reason to focus on the prevention and control of this disease. The selected biological fungicide, *Bacillus velezensis* strain BS023, was found to significantly inhibit the growth of *C. fioriniae*. Transcriptome analysis indicated that *B. velezensis* CFS initially damaged ribosomes, which then led to disturbance in genetic information processing, cell membranes, and energy metabolism in *C. fioriniae*. Additionally, treatment with *B. velezensis* CFS induced the metabolism of glutathione and the expression of ABC and MFS transporters as a compensatory response to the damage caused by *B. velezensis* CFS to *C. fioriniae*.

## Data availability statement

The datasets presented in this study can be found in online repositories. The names of the repository/repositories and accession number(s) can be found in the article/Supplementary material.

## Ethics statement

The manuscript presents research on animals that do not require ethical approval for their study.

## Author contributions

CF: Data curation, Investigation, Project administration, Validation, Writing – original draft. SW: Conceptualization, Investigation, Software, Writing – original draft. PY: Formal Analysis, Methodology, Project administration, Resources, Visualization, Writing – original draft. XZ: Data curation, Methodology, Supervision, Validation, Writing – review & editing. YY: Data curation, Formal analysis, Methodology, Project administration, Validation, Writing – review & editing, Investigation. SJ: Formal analysis, Investigation, Methodology, Project administration, Resources, Supervision, Writing – review & editing. HA: Formal analysis, Supervision, Writing – review & editing.
