# Supplementary material for: Identification of the Ilex macrocarpa anthracnose pathogen and the antifungal potential of the cell-free supernatant of Bacillus velezensis against Colletotrichum fioriniae
Source: Front Microbiol. 2024 Jun 20;15:1419436. doi: 10.3389/fmicb.2024.1419436 (PMC11222323; doi:10.3389/fmicb.2024.1419436)
Supplement: Supplementary file 1 [file Table_1.DOCX]

Supplementary Material

Supplementary Table S1| Sequences of Primers

| Gene | Upstream primer**（5^’^-3^’^）** | Downstream primer**（5^’^-3^’^）** |
| --- | --- | --- |
| ITS^a^ | TCCGTAGGTGAACCTGCGG | TCCTCCGCTTATTGATATGC |
| HIS3^a^ | AGGTCCACTGGTGGCAAG | AGCTGGATGTCCTTGGACTG |
| CHS-1^a^ | TGGGGCAAGGATGCTTGGAAGA | TGGAAGAACCATCTGTGAGAGTTG |
| ACT^a^ | ATGTGCAAGGCCGGTTTCG | TACGAGTCCTTCTGGCCCAT |
| TUB2^a^ | GGTAACCAAATCGGTGCTGCTTC | ACCCTCAGTGTAGTGACCCTTGGC |
| GAPDH^a^ | GCCGTCAACGACCCCTTCATTGA | GGGTGGAGTCGTACTTGAGCATGT |

^a^ Primers are ITS1/ITS4 (White et al. 1990), CYLH3F/CYLH3R (Crous et al. 2004), CHS-79F/CHS-345R (Weir et al. 2012), ACT512F/ACT, BT2A/BT2B (Carbone et al. 1999), and GDF/GDR (Liu et al. 2015), respectively.

Supplementary Table S2| Sequences used for the phylogenetic analyses

| species | strain | ITS | TUB2 | GAPDH | ACT | CHS-1 | HIS3 |
| --- | --- | --- | --- | --- | --- | --- | --- |
| *C.cosmi** | CBS 853.73 | JQ948274 | JQ949925 | JQ948604 | JQ949595 | JQ948935 | JQ949265 |
| *C. walleri** | CBS 125472 | JQ948275 | JQ949926 | JQ948605 | JQ949596 | JQ948936 | JQ949266 |
| *C.nymphaeae** | CBS 515.78 | JQ948197 | JQ949848 | JQ948527 | JQ949518 | JQ948858 | JQ949188 |
| *C.guajave** | IMI 350839 | JQ948270 | JQ949921 | JQ948600 | JQ949591 | JQ948931 | JQ949261 |
| *C.scovillei** | CBS 126529 | JQ948267 | JQ949918 | JQ948597 | JQ949588 | JQ948928 | JQ949258 |
| *C.lacticiphilum** | CBS 112989 | JQ948289 | JQ949940 | JQ948619 | JQ949610 | JQ948950 | JQ949280 |
| *C.brisbanense** | CBS 292.67 | JQ948291 | JQ949942 | JQ948621 | JQ949612 | JQ948952 | JQ949282 |
| *C.indonesiense** | CBS 127551 | JQ948288 | JQ949939 | JQ948618 | JQ949609 | JQ948949 | JQ949279 |
| *C.paxtonii** | IMI 165753 | JQ948285 | JQ949936 | JQ948615 | JQ949606 | JQ948946 | JQ949276 |
| *C.simmondsii** | CBS 122122 | JQ948276 | JQ949927 | JQ948606 | JQ949597 | JQ948937 | JQ949267 |
| *C.sloanei** | IMI 364297 | JQ948287 | JQ949938 | JQ948617 | JQ949608 | JQ948948 | JQ949278 |
| *C.chrysanthemi* | IMI 364540 | JQ948273 | JQ949924 | JQ948603 | JQ949594 | JQ948934 | JQ949264 |
| *C.costaricense** | CBS 330.75 | JQ948180 | JQ949831 | JQ948510 | JQ949501 | JQ948841 | JQ949171 |
| *C.limetticola** | CBS 1144 | JQ948193 | JQ949844 | JQ948523 | JQ949514 | JQ948854 | JQ949184 |
| *C.tamarilloi** | CBS 129814 | JQ948184 | JQ949835 | JQ948514 | JQ949505 | JQ948845 | JQ949175 |
| *C.lupini* | CBS 109225 | JQ948155 | JQ949806 | JQ948485 | JQ949476 | JQ948816 | JQ949146 |
| *C.cuscutae** | IMI 304802 | JQ948195 | JQ949846 | JQ948525 | JQ949516 | JQ948856 | JQ949186 |
| *C.melonis** | CBS 159.84 | JQ948194 | JQ949845 | JQ948524 | JQ949515 | JQ948855 | JQ949185 |
| *C.fioriniae** | CBS 128517 | JQ948292 | JQ949943 | JQ948622 | JQ949613 | JQ948953 | JQ949283 |
| *C.acutatum** | CBS 112996 | JQ005776 | JQ005860 | JQ948677 | JQ005839 | JQ005797 | JQ005818 |
| *C.acerbum** | CBS 128530 | JQ948459 | JQ950110 | JQ948790 | JQ949780 | JQ949120 | JQ949450 |
| *C.rhombiforme** | CBS 129953 | JQ948457 | JQ950108 | JQ948788 | JQ949778 | JQ949118 | JQ949448 |
| *C.phormii** | CBS 118194 | JQ948446 | JQ950097 | JQ948777 | JQ949767 | JQ949107 | JQ949437 |
| *C.kinghornii** | CBS 198.35 | JQ948454 | JQ950105 | JQ948785 | JQ949775 | JQ949115 | JQ949445 |
| *C.australe** | CBS 116478 | JQ948455 | JQ950106 | JQ948786 | JQ949776 | JQ949116 | JQ949446 |
| *C.salicis** | CBS 607.94 | JQ948460 | JQ950111 | JQ948791 | JQ949781 | JQ949121 | JQ949451 |
| *C.johnstonii** | CBS 128532 | JQ948444 | JQ950095 | JQ948775 | JQ949765 | JQ949105 | JQ949435 |
| *C.pyricola** | CBS 128531 | JQ948445 | JQ950096 | JQ948776 | JQ949766 | JQ949106 | JQ949436 |
| *C.godetiae** | CBS 133.44 | JQ948402 | JQ950053 | JQ948733 | JQ949723 | JQ949063 | JQ949393 |
| *C.orchidophilum** | CBS 632.80 | JQ948151 | JQ949802 | JQ948481 | JQ949472 | JQ948812 | JQ949142 |
| *C.fioriniae^a^* | XLHR17 | ON714636 | OQ025282 | OQ025279 | OQ025278 | OQ025281 | OQ025283 |
| *C.fioriniae* | KACC 48794 | MN960558 | MN974143 | MN974139 | MN974142 | MN974140 | MN974141 |
| *C.fioriniae* | EA7 | KY695261 | KY695260 | KY695258 | KY695256 | KY695257 | KY695259 |

^a^ The isolate used in the present study. Ex-Type (ex-epitype) strains are marked with an *.

Supplementary Table S3| Sequences used for the phylogenetic analyses

| Gene ID | Describtion | Upstream primer**（5^’^-3^’^）** | Downstream primer**（5^’^-3^’^）** |
| --- | --- | --- | --- |
| COL516b_002521 | ERG1 | GCCAGAAGAAGGACGACTATTT | GAGGGCGTAGAACTTTGACTT |
| COL516b_006134 | CYB5 | CCTCTGGAGCTTGATCGAATAC | GAGAAGGAAGTGTGCAGTGATA |
| COL516b_010027 | AMY | AACGAGAAGACGGCAATCTATAA | GATCAGAACCCATCAGGAAGTC |
| COL516b_005912 | ATG17 | AGGAGGCTGTTGTCTTGTATG | GGCATCGTAGGTTCTCTTCAG |
| COL516b_012101 | ATG8 | GGAGAAGGCCATCTTCATCTT | GCCGGAGTAGGTGATGTAAAG |
| COL516b_004489 | GNAT | GGTCGTCTATCTGCATATCTTGG | CGAACAGCTTCCTGCACTAT |
| COL516b_008851 | ACAD | GAGACTGAGGCCATGCTAAA | GAGTACGACAGACCACCAAAG |
| COL516b_010611 | ADH | TCCACGCTACCTCTCTTAACTA | CCATCGCCGAGTGGAATTAT |
| COL516b_006075 | ADH | AGCAATGGGCTCAGGTTATC | ACACCGGAGTACTTGACATTG |
| COL516b_002308 | GST | GCAGAGACTGAAGGAAGAGAAG | ACGAGTAGACGTAGCCGTAA |
| COL516b_004666 | MBL | ACGGCTGATGGGAAGATTG | CGTTGTTCCGTCCTCGTTAT |
| COL516b_007243 | ALDH | CTGCTACAACATGCTCTCCTAC | GGACTTGTTCTGGGTGTAGTTT |
| COL516b_002441 | GFA | AGGATGACCTCTCGACGTATAA | GGGTGTGAGGCTCGTATTTAG |
| COL516b_005616 | GFA | GGCGCTGTTCAACTATCATTTC | CAGAACATGGAGGAGGAGATTT |
| COL516b_006882 | GAT | CTGATTCTGGGCTCGTCTTATC | GGTAGTGTCGCCCTGTAATATG |
| COL516b_006820 | Qns1 | CTCCGTCCGTTCTTGTATGTC | CCCTATCCGCAATCTTCTTCTC |
| COL516b_001442 | PGD | GAGAAGCACTACCCTCTCTTTAC | CAGTTCTCCGAAGTAGACACTAAG |
| COL516b_003024 | MAPEG | CTTTGAGACGCTCGGTTTCTA | GCGGACGTAGATGATGTTGTA |
| COL516b_010985 | Grx | AAAGGTTCAGTCCCTCATCG | GGTGTAGTCTGCGTTGATCT |
| COL516b_008584 | TRR | AGCTCATGGAGAACATGAAGG | GAACTCGGTCGAGAACTTGAA |
| COL516b_007025 | POD | GGCTCGTCAACTGGTGTATT | GAAGCCCTCGTAGACATCTTG |
| COL516b_010810 | LETM1 | TCGACCTACCCGAGAAGAAA | GTTGGAGAAGACGGCTTTGA |
| COL516b_005988 | MRBS27/S33 | CAAGGATGTGCAGAGAGAGTT | CTCGGTCTTCGTCTTCTTCTTT |
| COL516b_003158 | RPL | GGTCAAGTCCACCATCAAGT | AGCAGAGAGACGAGGTAGTT |
| COL516b_003688 | Actin3 | GGCCGCAATCTTCTCCATAA | CATTGCCGTAGCAAGAGATAGA |

Supplementary Table S4| Transcriptome statistics of *Penicillium olsonii* WHG5.

| Sample name | TJCK | TJTM |
| --- | --- | --- |
| **Evaluation statistics** |  | |
| Number of clean reads | 32,816,930 | 31,681,566 |
| Clean bases (G) | 4.85 | 4.67 |
| Clean reads (%) | 95.59 | 95.72 |
| Numbers of clean reads total mapped to genome | 31,258,431 | 30,275,320 |
| Numbers of clean reads multiple mapped to genome | 1,152,606 | 1,112,054 |
| Numbers of clean reads uniquely mapped to genome | 30,105,825 | 29,163,266 |

Supplementary Table S5| DEGs involved in the oxidation‒reduction process

| Gene ID | Description | Treatment | Control | Log FC |
| --- | --- | --- | --- | --- |
| COL516b_008570 | Alcoholdehydrogenase | 51.40 | 23.81 | 1.11 |
| COL516b_000273 | Alcohol dehydrogenase | 199.29 | 93.54 | 1.09 |
| COL516b_010611 | Alcohol dehydrogenase | 456.42 | 0.14 | 11.63 |
| COL516b_006309 | Alcohol dehydrogenase | 269.30 | 9.03 | 4.90 |
| COL516b_001855 | Alcohol dehydrogenase | 394.31 | 13.64 | 4.85 |
| COL516b_001514 | Alcohol dehydrogenase | 2514.15 | 143.08 | 4.14 |
| COL516b_003853 | Alcohol dehydrogenase | 94.75 | 6.43 | 3.88 |
| COL516b_000996 | Alcohol dehydrogenase | 226.87 | 16.12 | 3.81 |
| COL516b_002590 | Alcohol dehydrogenase | 430.49 | 34.11 | 3.66 |
| COL516b_011360 | Alcohol dehydrogenase | 197.83 | 22.36 | 3.14 |
| COL516b_008956 | Alcohol dehydrogenase | 370.71 | 45.76 | 3.02 |
| COL516b_004296 | Alcohol dehydrogenase | 111.10 | 24.18 | 2.20 |
| COL516b_011404 | Alcohol dehydrogenase | 449.38 | 7.32 | 5.94 |
| COL516b_001478 | Alcohol dehydrogenase | 77.99 | 6.53 | 3.58 |
| COL516b_001893 | Alcohol dehydrogenase | 591.05 | 52.06 | 3.50 |
| COL516b_001796 | Alcohol dehydrogenase | 151.86 | 12.38 | 3.62 |
| COL516b_002336 | Alcohol dehydrogenase | 7.39 | 166.47 | -4.49 |
| COL516b_006075 | Alcohol dehydrogenase | 516.47 | 3249.33 | -2.65 |
| COL516b_011291 | Alcohol dehydrogenase | 78.75 | 196.60 | -1.32 |
| COL516b_001315 | Alcohol dehydrogenase | 211.68 | 449.78 | -1.09 |
| COL516b_009758 | Alcohol dehydrogenase | 30.83 | 145.74 | -2.24 |
| COL516b_003986 | Alcohol dehydrogenase | 10.43 | 73.90 | -2.82 |
| COL516b_006020 | Alcohol dehydrogenase | 21.77 | 56.04 | -1.36 |
| COL516b_011324 | Alcohol dehydrogenase | 88.78 | 626.18 | -2.82 |
| COL516b_004536 | Acyl-CoA dehydrogenase | 137.79 | 38.26 | 1.85 |
| COL516b_011438 | Acyl-CoA dehydrogenase | 89.35 | 18.55 | 2.27 |
| COL516b_010219 | Acyl-CoA dehydrogenase | 75.13 | 15.82 | 2.25 |
| COL516b_003666 | Acyl-CoA dehydrogenase | 152.04 | 37.80 | 2.01 |
| COL516b_008851 | Acyl-CoA dehydrogenase | 699.91 | 328.84 | 1.09 |
| COL516b_007755 | Acetyltransferase (GNAT) | 172.70 | 51.17 | 1.75 |
| COL516b_004482 | Acetyltransferase (GNAT) | 122.37 | 36.87 | 1.73 |
| COL516b_010367 | Acetyltransferase (GNAT) | 979.72 | 20.17 | 5.60 |
| COL516b_005439 | Acetyltransferase (GNAT) | 225.86 | 14.49 | 3.96 |
| COL516b_004489 | Acetyltransferase (GNAT) | 2024.42 | 143.07 | 3.82 |
| COL516b_011603 | Acetyltransferase (GNAT) | 187.73 | 21.19 | 3.15 |
| COL516b_003889 | Acetyltransferase (GNAT) | 66.71 | 12.05 | 2.47 |
| COL516b_008126 | Acetyltransferase (GNAT) | 516.43 | 207.03 | 1.32 |

Supplementary Table S6| DEGs related to membranes

| Gene ID | Description | Treatment | Control | Log FC |
| --- | --- | --- | --- | --- |
| COL516b_003024 | MAPGE | 9.77 | 525.87 | -5.75 |
| COL516b_006897 | CFEM | 3.75 | 52.01 | -3.79 |
| COL516b_006713 | CFEM | 24.94 | 301.57 | -3.60 |
| COL516b_004099 | CFEM | 7.87 | 83.76 | -3.41 |
| COL516b_005495 | phosphate transporter | 29.88 | 200.43 | -2.75 |
| COL516b_005336 | Ferric reductase like transmembrane component | 10.60 | 69.09 | -2.70 |
| COL516b_008031 | Predicted membrane protein | 18.99 | 112.67 | -2.57 |
| COL516b_006145 | CFEM | 9.63 | 54.07 | -2.49 |
| COL516b_010939 | Plasma membrane ATPase | 37.17 | 205.97 | -2.47 |
| COL516b_010843 | Mitochondrial import inner membrane translocase subunit | 269.21 | 1134.08 | -2.07 |
| COL516b_005668 | Ferric reductase like transmembrane | 14.54 | 51.06 | -1.81 |
| COL516b_004213 | Mitochondrial import receptor | 22.44 | 77.62 | -1.79 |
| COL516b_011119 | Lung seven transmembrane receptor | 23.99 | 78.70 | -1.71 |
| COL516b_009823 | MAPGE | 26.36 | 85.48 | -1.70 |
| COL516b_008901 | Ferric reductase like transmembrane | 27.50 | 87.71 | -1.67 |
| COL516b_006373 | CFEM | 17.22 | 52.50 | -1.61 |
| COL516b_012057 | Tim17/Tim22/Tim23/Pmp24 family | 38.72 | 117.77 | -1.60 |
| COL516b_002912 | Cation-transporting ATPase | 25.55 | 73.66 | -1.53 |
| COL516b_002488 | COPI associated protein | 97.42 | 260.86 | -1.42 |
| COL516b_011080 | Tim17/Tim22/Tim23/Pmp24 family | 42.85 | 111.58 | -1.38 |
| COL516b_009501 | Transmembrane alpha-helix domain-containing protein | 87.52 | 225.75 | -1.37 |
| COL516b_005590 | Inositol phospholipid synthesis protein Scs3p | 46.93 | 120.47 | -1.36 |
| COL516b_001005 | CFEM | 38.94 | 97.94 | -1.33 |
| COL516b_012249 | Mitochondrial inner membrane protein | 52.00 | 117.76 | -1.18 |
| COL516b_001289 | Tim44-like domain | 36.56 | 77.99 | -1.09 |
| COL516b_012522 | ferric reductase like transmembrane component | 57.35 | 115.16 | -1.01 |
| COL516b_009947 | CFEM | 122.78 | 41.58 | 1.56 |
| COL516b_000080 | phospholipid-translocating P-type ATPase | 61.87 | 23.85 | 1.38 |
| COL516b_012417 | Ferric reductase like transmembrane component | 309.58 | 130.00 | 1.25 |
| COL516b_007485 | LMBR1-like membrane protein | 81.62 | 37.14 | 1.14 |

Supplementary Table S7| DEGs related to ribosome metabolism

| Gene ID | Description | Treatment | Control | Log FC |
| --- | --- | --- | --- | --- |
| COL516b_010810 | LETM1 | 26.63 | 164.02 | -2.62 |
| COL516b_003511 | RPSS9/S16 | 20.43 | 62.46 | -1.61 |
| COL516b_002981 | MRPL | 21.02 | 61.77 | -1.55 |
| COL516b_003559 | SPB1 | 36.60 | 105.28 | -1.52 |
| COL516b_007501 | RPL7/12 | 68.01 | 193.48 | -1.51 |
| COL516b_007971 | RPL12 | 33.26 | 92.46 | -1.47 |
| COL516b_008198 | RPL28 | 33.32 | 92.05 | -1.47 |
| COL516b_005561 | RPL | 28.80 | 79.10 | -1.46 |
| COL516b_005344 | RPL | 83.99 | 219.36 | -1.39 |
| COL516b_002579 | MRPL | 77.96 | 195.05 | -1.32 |
| COL516b_007325 | RPL16p | 30.58 | 75.68 | -1.31 |
| COL516b_009494 | RPLl13 | 21.18 | 51.76 | -1.29 |
| COL516b_008412 | RPS15 | 44.77 | 106.98 | -1.26 |
| COL516b_003452 | ADK | 41.26 | 94.97 | -1.20 |
| COL516b_005988 | MRBS27/S33 | 499.66 | 1144.55 | -1.20 |
| COL516b_004650 | RPL | 98.03 | 219.84 | -1.17 |
| COL516b_006546 | LETM1 | 186.48 | 410.69 | -1.14 |
| COL516b_000671 | RPL25 | 76.71 | 168.21 | -1.13 |
| COL516b_011773 | RPL11 | 139.31 | 303.78 | -1.12 |
| COL516b_004793 | RPS8 | 25.97 | 55.65 | -1.10 |
| COL516b_012363 | RPS10 | 128.77 | 272.88 | -1.08 |
| COL516b_010318 | RPL | 29.42 | 62.05 | -1.08 |
| COL516b_009065 | RPL | 35.12 | 73.85 | -1.07 |
| COL516b_007481 | EIF-6 | 918.29 | 66.44 | 3.79 |
| COL516b_011073 | SBDS | 860.53 | 113.90 | 2.92 |
| COL516b_010093 | FER4 | 212.02 | 40.65 | 2.38 |
| COL516b_004336 | RPL | 1071.39 | 230.50 | 2.22 |
| COL516b_003158 | RPL | 1586.65 | 488.62 | 1.70 |
| COL516b_004220 | CLD | 205.40 | 94.98 | 1.11 |

Supplementary Table S8| DEGs in ABC transportor family

| Gene ID | Description | Treatment | Control | Log FC |
| --- | --- | --- | --- | --- |
| COL516b_001826 | ABC | 423.41 | 13.02 | 5.02 |
| COL516b_011937 | ABC2 | 62.68 | 2.00 | 4.97 |
| COL516b_006528 | ABC2 | 77.17 | 4.66 | 4.05 |
| COL516b_003720 | ABC | 886.74 | 75.76 | 3.55 |
| COL516b_003719 | ABC | 261.71 | 22.52 | 3.54 |
| COL516b_007704 | ABC | 634.81 | 82.88 | 2.94 |
| COL516b_009262 | ABC | 66.44 | 15.29 | 2.12 |
| COL516b_006498 | ABC | 232.51 | 89.21 | 1.38 |
| COL516b_003361 | ABC2 | 175.84 | 79.64 | 1.14 |
| COL516b_000407 | ABC | 1.34 | 59.67 | -5.48 |
| COL516b_009845 | ABC | 21.03 | 56.59 | -1.43 |
| COL516b_005158 | ABC | 20.25 | 52.25 | -1.37 |

Supplementary Table S9| DEGs in MFS transportor family

| Gene ID | Description | Treatment | Control | Log FC |
| --- | --- | --- | --- | --- |
| COL516b_004727 | MFS transportor | 60.81 | 0.00 | 13.91 |
| COL516b_008466 | MFS transportor | 50.95 | 0.24 | 7.69 |
| COL516b_001166 | MFS transportor | 79.76 | 0.73 | 6.76 |
| COL516b_006631 | MFS transportor | 288.69 | 3.57 | 6.34 |
| COL516b_004540 | MFS transportor | 162.63 | 3.60 | 5.50 |
| COL516b_006243 | MFS transportor | 73.13 | 4.15 | 4.14 |
| COL516b_006813 | MFS transportor | 1613.15 | 94.98 | 4.09 |
| COL516b_009759 | MFS transportor | 188.52 | 11.59 | 4.02 |
| COL516b_001758 | MFS transportor | 66.12 | 6.48 | 3.35 |
| COL516b_005169 | MFS transportor | 304.98 | 31.86 | 3.26 |
| COL516b_009710 | MFS transportor | 91.36 | 9.85 | 3.21 |
| COL516b_009711 | MFS transportor | 99.98 | 14.20 | 2.81 |
| COL516b_005789 | MFS transportor | 65.51 | 13.00 | 2.33 |
| COL516b_007718 | MFS transportor | 206.64 | 63.00 | 1.71 |
| COL516b_003021 | MFS transportor | 190.64 | 70.91 | 1.43 |
| COL516b_008390 | MFS transportor | 145.92 | 61.47 | 1.25 |
| COL516b_000287 | MFS transportor | 192.29 | 91.41 | 1.07 |
| COL516b_001771 | MFS transportor | 0.00 | 64.48 | -13.85 |
| COL516b_009409 | MFS transportor | 6.01 | 124.61 | -4.37 |
| COL516b_012297 | MFS transportor | 12.71 | 244.83 | -4.27 |
| COL516b_008944 | MFS transportor | 45.43 | 847.94 | -4.22 |
| COL516b_002010 | MFS transportor | 4.37 | 59.19 | -3.76 |
| COL516b_006484 | MFS transportor | 35.96 | 311.23 | -3.11 |
| COL516b_001452 | MFS transportor | 22.75 | 174.33 | -2.94 |
| COL516b_000984 | MFS transportor | 23.26 | 175.92 | -2.92 |
| COL516b_006226 | MFS transportor | 7.81 | 56.06 | -2.84 |
| COL516b_005281 | MFS transportor | 9.56 | 59.25 | -2.63 |
| COL516b_001543 | MFS transportor | 41.66 | 210.73 | -2.34 |
| COL516b_010956 | MFS transportor | 28.11 | 97.68 | -1.80 |
| COL516b_011334 | MFS transportor | 59.05 | 155.32 | -1.40 |
| COL516b_003293 | MFS transportor | 47.93 | 123.48 | -1.37 |
| COL516b_011501 | MFS transportor | 105.97 | 249.08 | -1.23 |
| COL516b_011940 | MFS transportor | 78.17 | 166.37 | -1.09 |
| COL516b_002201 | MFS transportor | 48.00 | 96.99 | -1.01 |
